# Regulatory T cell epitope content in human antibodies decreases during maturation

**DOI:** 10.3389/fimmu.2025.1535826

**Published:** 2025-04-17

**Authors:** Andres H. Gutierrez, Frances E. Terry, Amy S. Rosenberg, William D. Martin, Anne S. De Groot

**Affiliations:** EpiVax, Inc., Providence, RI, United States

**Keywords:** B cell maturation, germinal center (GC), somatic hypermutation (SHM), antibody repertoire, B cell receptor (BCR), HLA-DR T cell epitopes, Tregitopes, regulatory T cell epitopes

## Abstract

**Introduction:**

Antibody maturation in the lymphoid follicle produces antibodies with improved binding affinity. This process requires iterative rounds of mutation and B cell expansion, supported by T cells that recognize epitopes presented on the B cell’s MHC-II. In this comprehensive antibody repertoire analysis, we find that established regulatory T cell epitopes (Tregitopes) decline in maturing antibody sequences as somatic hypermutation (SHM) increases, but potential T effector epitopes do not decline. A previous analysis of B cell receptor (BCR)-derived HLA-DR epitopes present in memory antibody repertoires from seven healthy human donors revealed a decrease in donor-specific epitope content with SHM. Moreover, T cell epitope depletion was associated with class-switching and long-term secretion of antibody into serum. Significant depletion of high-affinity germline-encoded epitopes in high SHM sequences was also observed, but the predicted phenotype of T cells responding to the BCR-derived epitopes (regulatory vs. effector) was not previously evaluated.

**Methods:**

In this follow-on study, we screened a different set of four donor repertoires to investigate the dynamics of donor-specific HLA-DR T cell epitopes and three subsets of T cell epitope content: previously validated T cell epitopes recognized by thymus-derived Tregs (Tregitopes), potentially tolerated T cell epitopes, and potential effector T cell epitopes.

**Results:**

Our results show that Tregitope content reduction is correlated with SHM, suggesting that Tregitopes are removed during maturation. Moreover, T cell epitopes that are likely to be tolerated or tolerogenic were also removed with SHM progression. In contrast, potential T effector epitope content increased with SHM. Tregitope depletion occurred in multiple V-gene pair combinations and was the most frequent T cell epitope change. Furthermore, Tregitope content in IgA and IgG sequences was lower and had greater negative correlation with SHM than IgM, indicating that Tregitope removal is likely associated with class-switching. Tregitope depletion was also associated with maturation to plasmablasts. In vitro, representative Tregitopes inhibited CD4+ T cell proliferation. Mutations introduced by SHM altered Tregitope HLA-DR binding affinities.

**Discussion:**

The correlation of Tregitope depletion with increasing SHM implies that the activity of thymus-derived Treg cells in immune responses to antibodies is diminished with SHM, maturation, and isotype switching, supporting the generation of anti-idiotype responses.

## 1 Introduction

The development of effective humoral immune response requires both a high degree of B cell diversity, allowing for recognition of a wide variety of antigenic structures, and an ability to generate antibodies with increased binding affinity. Random recombination of V (variable), D (diversity), and J (joining) genes in the bone marrow produces massive B cell receptor (BCR) repertoire diversity (1, 2). Upon antigen exposure, affinity maturation by somatic hypermutation (SHM) produces antibodies with improved binding to their cognate antigen, which is critical to the development of an efficient functional adaptive immune response (3, 4). While the proactive production of antibodies that have high affinity to target antigens is critical for the development of immunity, it is also costly in terms of energy expended and for the potential to generate autoantibodies as a by-product of the process. Therefore, it is important (in terms of energy expenditure) for this process to be well regulated and only initiated when necessary.

**Figure 1:**
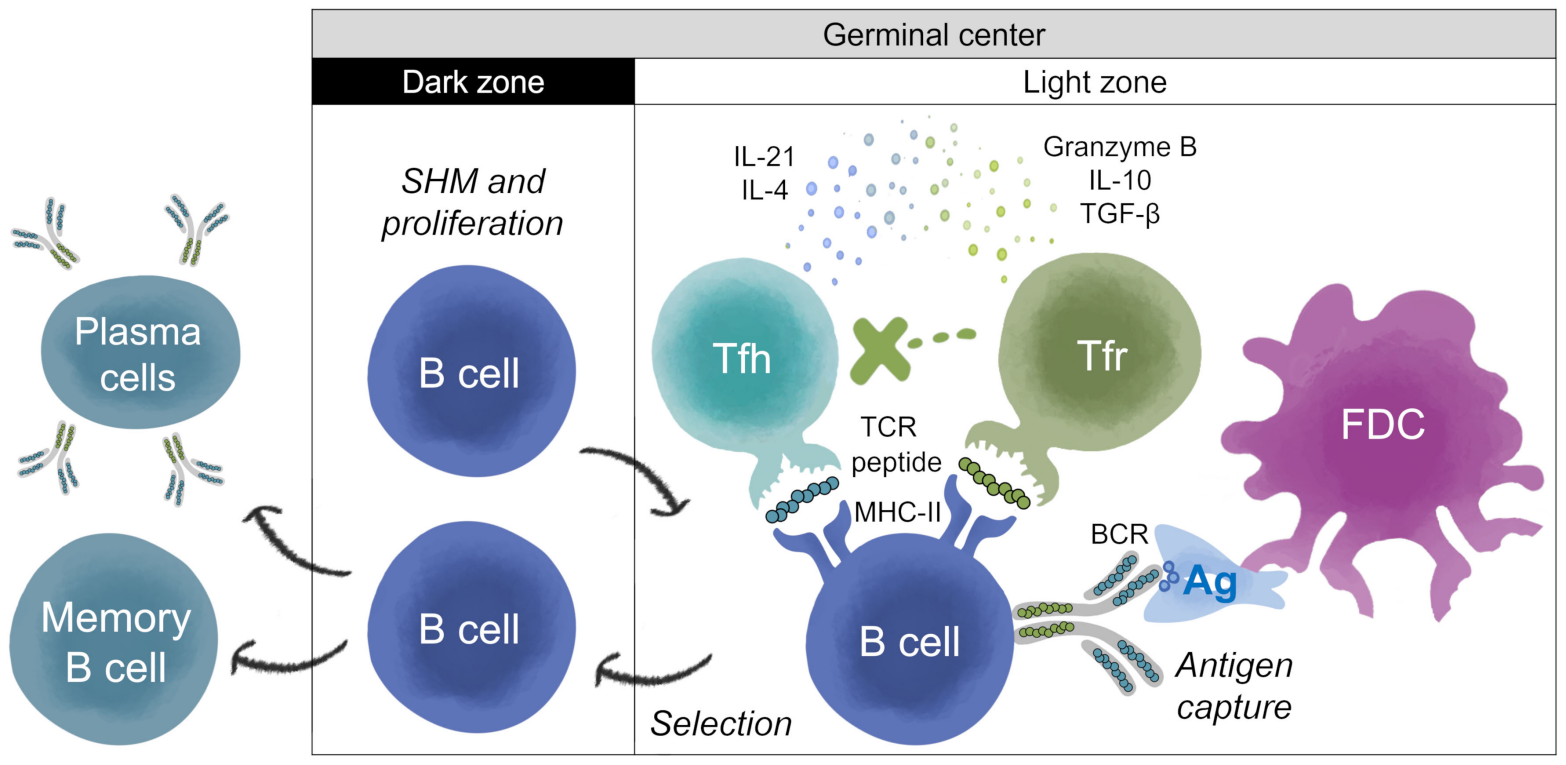
T help and Treg regulation of antibody development in germinal center. Adapted from Stebegg et al. (7). The germinal center (GC) is divided into two zones; the dark zone (DZ) and the light zone (LZ). Somatic Hypermutation (SHM) and B cell proliferation occur in the DZ. Antigen (Ag) processing and presentation, and regulation of the B cell response, takes place in the LZ. In the LZ, B cells known as centrocytes compete for antigen captured by follicular dendritic cells (FDCs). GC B cells with higher affinity BCR can internalize and process BCR-antigen complexes into peptides that are loaded onto Major Histocompatibility Complex Class II (MHC-II) molecules for presentation to cognate T follicular helper (Tfh) cells to undergo positive selection. Peptide:MHC-II complexes displayed on the surface of B cells are recognized by Tfh T cell receptors (TCRs). Upon receiving positive survival signals from Tfh cells, centrocytes re-enter the DZ for further rounds of proliferation and SHM, after which they exit the GC as memory B cells or high-affinity antibody-secreting cells (plasmablasts and plasma cells), and some become long-lived plasma cells. Thymic-derived T follicular regulatory (Tfr) cells are also present in the LZ, and their main function is to restrain the development of B cell GC (16, 23). Tfr cells may be activated by regulatory T cell epitopes present in the antigen or in the sequences of the BCR, which is also processed and presented by the B cell (23, 58).Tfr cells prevent B cells from being activated by Tfh, most likely by secreting IL-10, granzyme B or TGF-β, and/or attenuating cytokine production (e.g., IL-21 and IL-4). However, in some settings, B and Tfh cells can overcome this suppression due to the presence of higher concentrations of Tfh cells, and/or other mechanisms. In settings where Tfh and B cells are able to escape Tfr cell suppression, GCs start to form (15). Tfr cells restrain B-T helper cell interactions, which is required for the feedback loop enabling GC B cells to survive (16). Tfr can act as a brake on B cell maturation, reducing their ability to progress through subsequent cycles of SHM, and ultimately reducing their conversion to B memory and plasma secreting cells. The overall results (B cell restraint or activation) may depend on the balance of the regulatory or activating signals.

During T cell-dependent antibody maturation, B cells bind to foreign antigens through their naïve membrane-bound immunoglobulin (Ig), known as BCR (5, 6). B cells then migrate from the B cell follicle to the border of the T cell zone where, depending on their BCR-antigen affinity, they begin to form germinal centers (GCs) with the support of antigen-specific CD4+ T helper cells (7). GCs are polarized structures where B cells undergo iterative cycles of clonal expansion and SHM of Ig variable region genes, followed by affinity-based selection, leading to proliferation and differentiation into memory B cells and antibody-secreting cells (plasmablasts and plasma cells), and some become long-lived plasma cells (8) (**Figure 1**). Engagement with antigen via their antigen-specific BCR and support from cognate CD4+ T cells also stimulates B cells to change the constant domain of the Ig heavy chain by class-switch recombination, which changes the expressed Ig isotype from IgM to IgG, IgA, or IgE, and determines the effector function of the antibodies (9). Both SHM and class-switch recombination require the enzyme activation-induced cytidine deaminase (AID) (9, 10).

T cells play a critical role in the GCs. CD4+ T helper cells in the lymphoid follicles mediate the selection of B cells with high-affinity BCRs in GCs. BCR-antigen complexes are endocytosed by B cells, processed into peptides, and loaded onto Major Histocompatibility Complex Class II (MHC-II) molecules for presentation to cognate T follicular helper (Tfh) cells (11) (**Figure 1**). Peptide:MHC-II complexes displayed on the surface of B cells are recognized by Tfh T cell receptors (TCRs). This interaction leads to secretion of cytokines and chemokines by Tfh that regulate the affinity maturation, clonal expansion, phenotype, and functional fate of the B cells (11, 12). In addition to Tfh cells, T follicular regulatory (Tfr) cells are also found in the B cell follicle and the GCs (12). Tfr cells are a subset of regulatory T (Treg) cells that originate from CD4+ CD25+ FoxP3+ precursors and express the GC-defining transcriptional factor Bcl6 (13, 14). Tfr cells regulate Tfh cells and B cell proliferation, suppress autoreactive and non-antigen specific GC B cells, maintain optimal antigen-specific humoral responses, influence cytokine production by Tfh cells and class-switching, thus playing a critical role in the GC response and antibody maturation (15–19). Thus, Tfr restrain antibody development, reducing energy expenditure, while Tfh promote highly targeted, high affinity antibody response.

While it is clear that T helper epitopes contained in antigen sequences play an important role in the development of high affinity BCRs, the BCR sequences themselves also contain T cell epitopes that may contribute to B cell maturation. In a previous study, Gutiérrez-González et al. evaluated the role of BCR-derived MHC-II epitopes in antibody selection during maturation (20). In that *in silico* analysis of T cell epitope content present in native antibody repertoires (paired variable heavy (VH) and variable light (VL) chain sequences) from healthy human donors, T cell epitope prediction was focused on human leukocyte antigen (HLA)-DR, one of three loci that encode MHC-II molecules. The results revealed a decrease in donor-specific, predicted T cell epitope content with SHM in class-switched antibodies, suggesting removal of HLA-DR-restricted T cell epitopes during antibody maturation. Moreover, certain heavy and light chain V-gene pairs showed more T cell epitope removal than others. Further investigation identified significant depletion of high-affinity germline-encoded epitopes in antibody regions subject to high rates of SHM. Some of these high-affinity epitopes were found in antibody frameworks and overlapped self-peptides that are commonly detected in MHC-II elution experiments. Although predicted T cell epitope phenotype was not investigated in this initial study, one of the germline peptides that was consistently lost in antibodies that underwent high SHM overlapped a previously identified and validated regulatory T cell epitope (Tregitope) (21). Our evaluation of the BCR repertoire in the Gutiérrez-González study suggested that most of the T cell epitopes that were depleted during SHM were Tregitopes.

Multiple Tregitopes have been identified within human antibody sequences (21, 22). Some of them are adjacent to the highly variable and potentially immunogenic complementarity-determining regions (CDR). They are generally numbered by their location in the germline sequence (e.g., Tregitope 9, 88 (VH), 167 and 289 (Fc)). In a recent study of IgG Fc, sequences corresponding to Tregitopes 167 and 289 were demonstrated to be both tolerogenic and present on the surface of B cells that were also IgG+ (23).

Tregitopes, as originally defined by De Groot et al., are recognized by thymus-derived Treg cells (tTregs), bind multiple HLA-DR molecules and promote tolerance by stimulating tTreg activity (22). *In vitro* and *in vivo* studies have shown that Tregitopes found in IgG activate and expand Tregs and promote induction of immunosuppressive cytokines (TGFβ and IL-10) (22, 24). Tregitope sequences are frequently found within monoclonal antibodies that elicit low levels of antidrug antibody (ADA) response; they are less frequently found in monoclonal antibodies that elicit higher levels of ADA response (25, 26).

To investigate further whether the T cell epitopes that are removed from BCRs during antibody maturation are Tregitopes or Tregitope-like, as we initially suspected when performing the analysis in the collaboration with Gutiérrez-González et al., we performed an *in silico* analysis of the dynamics of donor-specific HLA-DR T cell epitope content, Tregitopes, potentially tolerated T cell epitopes, and potential T effector epitopes, in the repertoire of natively paired VH and VL chains of memory BCRs studied by Jaffe et al. from donors with known HLA-DR alleles (27).

This, the resulting analysis of the relationship between donor-specific HLA-DR epitope content and molecular markers of antibody maturation mediated by T cells, namely SHM, isotype class-switching, and maturation to plasmablasts, showed that low Tregitope content in BCRs was associated with evidence of greater T cell help. We further evaluated the effect of representative VH Tregitopes (9A and 88) on CD4+ T cell proliferation and the effect of mutations introduced by SHM *in vitro*.

Our results are consistent with studies that have demonstrated a key role for Tfr in the modulation of antibody maturation in the lymph node (16, 28, 29) and they have potential implications for the design of improved protein-based therapeutics that might better evade the promotion of ADA responses. We have speculated that Tregitopes present in antibodies, by stimulating tTregs, may help to suppress, to some extent, anti-idiotype immune responses as well as immune responses to allergens and foreign antigens. The removal of Tregitopes in mature antibodies may also enable their removal by the “idiotypic network” at the termination of immune response to a foreign pathogen (30). Thus, the identification of Tregitope sequences in antibodies, and improved understanding of their natural function, is critically important for drug development and for immunology research.

## 2 Results

### 2.1 T cell epitope content, Tregitope content, and potentially tolerated T cell epitopes in BCRs decline with SHM while potential T effector epitope content increases

To investigate the dynamics of T cell epitope content in human antibody repertoires and its relationship with markers of antibody development, we screened previously published natively paired VH and VL sequences from four healthy human subjects (27) using EpiMatrix, a high-throughput HLA-DR epitope prediction algorithm (26). The analyzed database contains information on 249,958 paired heavy and light chain sequences of memory BCRs and the HLA-DR alleles of donors, which were non-identical.

First, we analyzed the relationship between SHM and donor-specific putative HLA-DR T cell epitope content, Tregitope content, potentially tolerated T cell epitopes, and potential T effector content. Using the EpiMatrix system, each complete antibody (paired VH and VL) was screened for potential donor-specific HLA-DR T cell epitopes. T cell epitope content was assessed as the sum of the scores of all donor-specific T cell epitopes. Individual donor-specific T cell epitopes were further classified as known Tregitopes, potentially tolerated T cell epitopes, or potential T effector epitopes. Using JanusMatrix (JMX), potentially tolerated (JMX high) and potential T effector epitopes (JMX low) were classified based on cross-conservation with human proteins (31). Nine-mer peptides are considered cross-conserved with a human-derived 9-mer peptide if both peptides are predicted to bind the same HLA and both peptides share TCR-facing residues (in the case of HLA-DR, relative binding positions 2, 3, 5, 7, and 8). Previous analysis suggested that T cell epitopes with higher degree of cross-conservation with human proteins were significantly more likely to be associated with IL-10 secretion or a null response (HLA binding without associated report of immune response) (32). Such T cell epitopes are likely to be at least tolerated by the human immune system and may be actively tolerogenic (33). Thus, T cell epitopes cross-conserved with three or more putative human T cell epitope 9-mers were classified as JMX high and considered potentially tolerated. T cell epitopes cross-conserved with less than three putative human T cell epitope 9-mers were classified as JMX low and considered potential T effector epitopes. Tregitope content, potentially tolerated T cell epitope (JMX high) content, and potential T effector (JMX low) content were calculated as the sum of EpiMatrix scores of donor-specific epitopes in each subset. Thus, the sum of the three subsets equals the total T cell epitope content.

**Figure 2:**
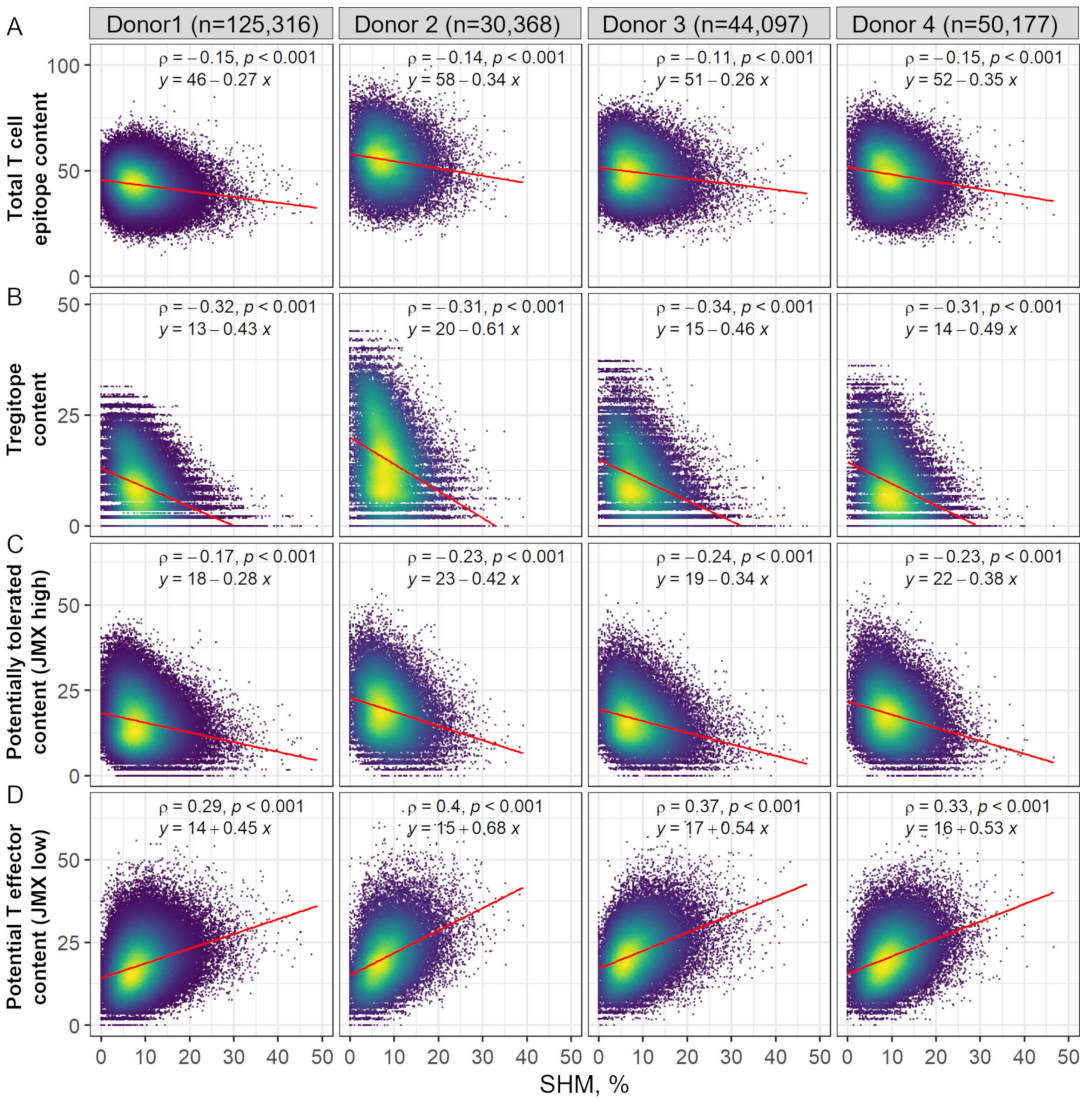
Donor-specific HLA-DR T cell epitope content, Tregitope content, and potentially tolerated T cell epitope content in antibody sequences decline with SHM, while potential T effector content increases. Scatter plot of **(A)** Total T cell epitope content and subsets of **(B)** Tregitope content, **(C)** potentially tolerated content (JMX high), and **(D)** potential T effector content (JMX low) vs. SHM. SHM percentages were calculated based on the identity percentage between heavy and light chain V-genes and their corresponding germlines using IgBLAST. Each point represents one antibody sequence; points are colored by data density from low (purple) to high (yellow). The number of antibodies per donor is shown at the top of the figure. Spearman ρ correlation and p-values are indicated. Linear regression equations and lines (red) are also shown.

Consistent with previously published results of T cell epitope content calculated for nine supertype HLA-DR alleles (20), T cell epitope content declined with SHM (**Figure 2A**). Donor-specific Tregitope content also declined with SHM (**Figure 2B**). Tregitope content had the greatest negative correlation with SHM. In addition, the slope of the regression line relating Tregitope content and SHM was greater than the slope of the line relating T cell epitope content with SHM, suggesting that the loss of T cell epitope content with SHM was mainly due to removal of Tregitope content. Similarly, JMX high T cell epitope content was negatively correlated with SHM (**Figure 2C**). Despite a lower correlation with SHM than Tregitope content, the loss of potentially tolerated (JMX high) epitopes also contributed to the overall loss of T cell epitope content. In contrast, JMX low, potential T effector epitope content, was positively correlated with SHM (**Figure 2D**). The increased positive slope of the regression lines for JMX low suggests that potential T effector content increased with SHM. Taken together, these results suggest that antibodies lose T cell epitope content and specifically, more Tregitopes, and potentially tolerated T cell epitopes with SHM while gaining potential T effector content. The overall loss of T cell epitope content with SHM was mainly due to removal of Tregitopes with a significant contribution from potentially tolerated T cell epitopes.

### 2.2 Isotype class-switching is correlated with removal of Tregitope content from BCRs

**Figure 3:**
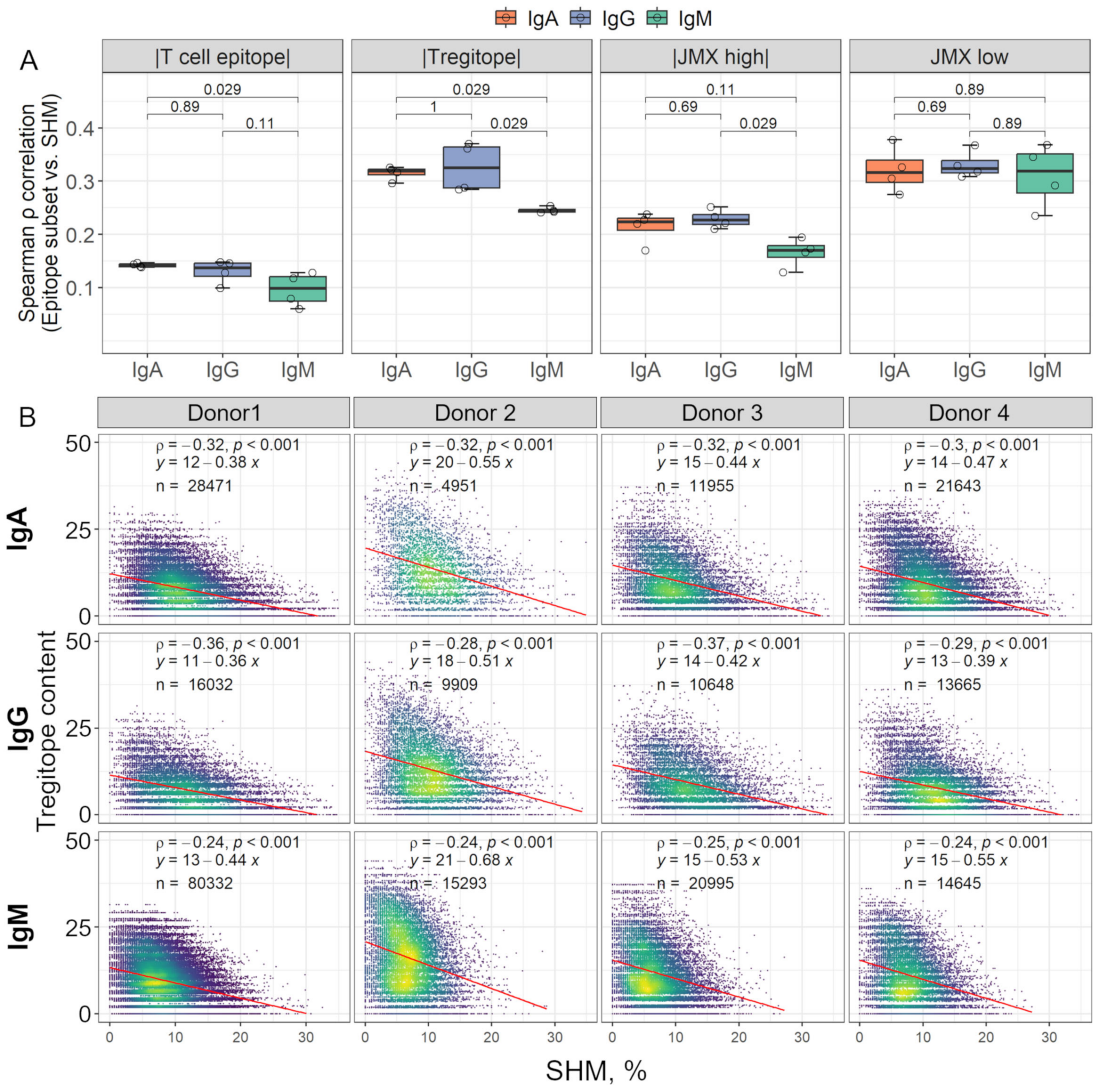
Isotype class-switching is (negatively) correlated with Tregitope content loss in complete antibodies. **(A)** The box plots show Spearman’s correlation coefficients (ρ) calculated between SHM and each T cell epitope content subset for all the donors. Correlation coefficients for individual donors are shown as open circles. For visualization, correlation coefficients for T cell epitope content, Tregitope content, and JMX high are shown as |absolute values|. Correlation coefficients were compared between isotypes using Wilcoxon rank sum test; p-values for each comparison are shown. **(B)** Scatter plots of Tregitope content vs. SHM by isotype. Each point represents one antibody sequence; points are colored by data density from low (purple) to high (yellow). The number of antibodies per isotype is shown in each plot. Spearman ρ correlation and p-values are indicated. Linear regression equations and lines (red) are also shown.

Since antibody class-switching, like SHM, is regulated and mediated by Tfr and Tfh responses, which requires TCR interaction with peptide:HLA-DR complexes on B cells, antibodies were grouped by isotype to evaluate the association between each T cell epitope content subset and class-switching. Overall, Tregitope content in the IgA and IgG antibody repertoires had the greatest (negative) correlation with SHM (**Figure 3**, **Supplementary Table 1**). Spearman ρ correlation coefficients between Tregitope content and SHM for IgA and IgG antibodies were significantly greater than coefficients for IgM antibodies (**Figure 3A**). IgA correlation coefficients between T cell epitope content and SHM were also significantly greater than those of IgM. For JMX high, IgG correlation coefficients were significantly greater than those of IgM. However, in both cases, T cell epitope content and JMX high correlations were weak compared to Tregitope content correlations. For JMX low content, although correlation coefficients were high, no significant differences were observed between isotypes. When binned by SHM, we observed differences in Tregitope content among isotypes for antibodies with similar SHM (**Supplementary Figure 1**). IgG and/or IgA Tregitope content was significantly lower than that of IgM, with one exception (donor 2, SHM 0-5). For three of the donors, IgG Tregitope content was lower than that of IgA. For potentially tolerated (JMX high) and potential T effector content (JMX low) differences among isotypes were not as consistently significant as those of Tregitope content. Considering antibody class-switching as a marker of T cell response, the stronger (negative) correlation observed between Tregitope content and SHM for class-switched IgA and IgG antibodies and lower Tregitope content compared to IgM for antibodies with similar SHM, indicates that class-switching is associated with Tregitope removal during antibody development.

### 2.3 Lower Tregitope content in BCRs is associated with maturation to plasmablasts

**Figure 4:**
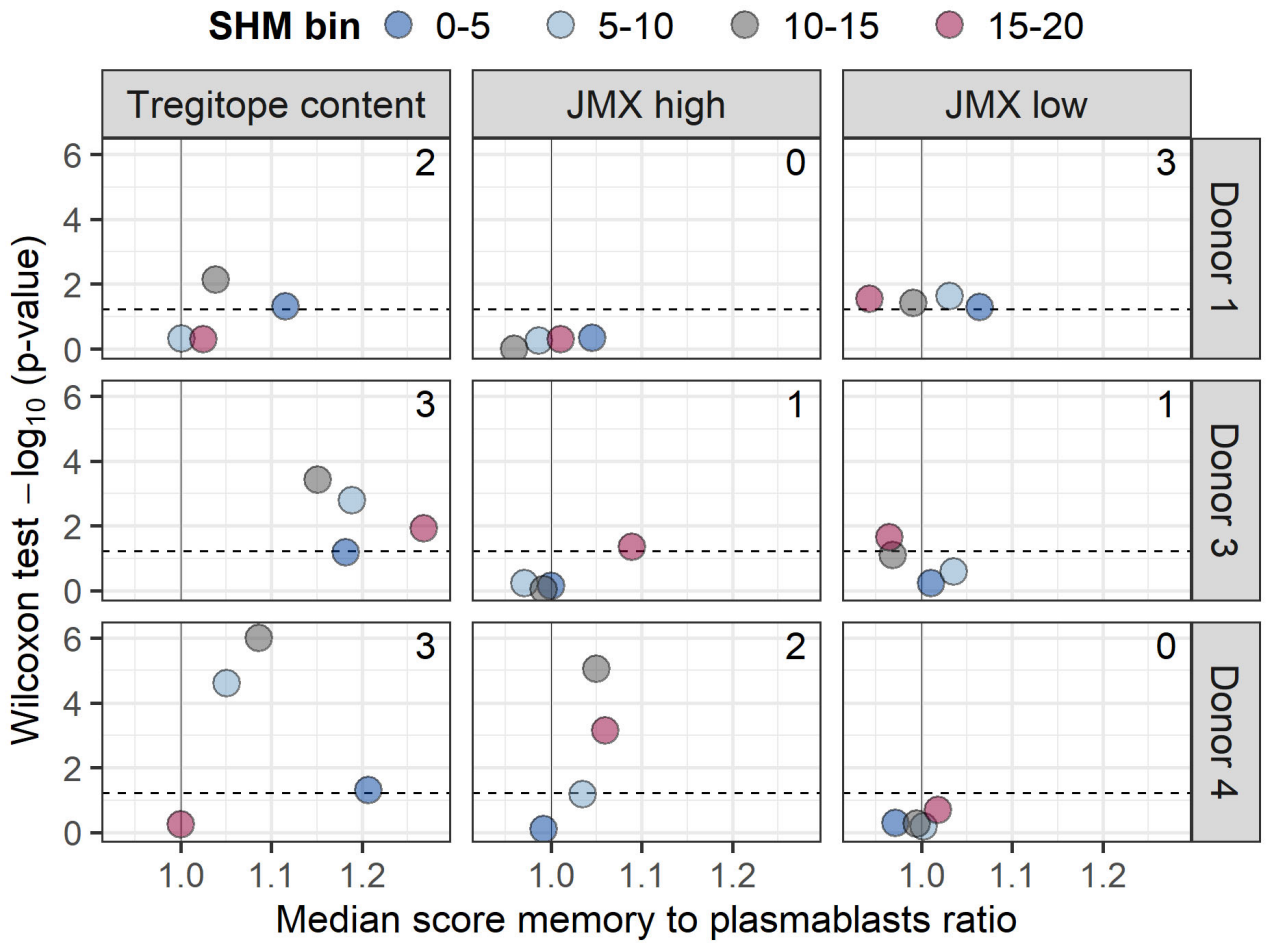
Maturation to plasmablasts is associated with lower Tregitope content. Isotype-switched memory B cells and plasmablasts for three donors (donors 1, 3, and 4) were binned based on SHM (truncated to 20%; 5% range; bins: 0-5, 5-10, 10-15, and 15-20). Tregitope, JMX high, and JMX content were compared within each SHM bin using a Wilcoxon rank sum test. Volcano plots of ratios of median Tregitope (left), JMX high (middle), and JMX low content (right) for memory B cells to plasmablasts for each SHM bin vs. Wilcoxon rank sum test -log_10_ (p-values) are shown for each donor. Ratio>1 indicates lower content in plasmablasts. The number of statistically significant observations is shown in each panel. P-value for donor 4, SHM 10-15 was zero; it was set to 0.000001 to calculate -log_10_ value.

In addition to mediating SHM and antibody class-switching, T cells provide stimulatory signals for B cells to transition to plasmablasts which, along with long-lived plasma cells, secret serum antibodies. To investigate whether any of the subsets of T cell epitope content studied here could be associated with B cell maturation to plasmablasts, we compared Tregitope, JMX high, and JMX low content predicted in isotype-switched BCR sequences of memory B cells and plasmablasts. In addition to BCRs of memory B cell sequences analyzed above, paired VH and VL BCR sequences of plasmablasts were available for three donors. Plasmablasts were screened using the same approach applied to memory B cells. Memory B cells and plasmablasts were binned based on SHM. For each SHM bin, Tregitope, JMX high, and JMX low content was compared between memory B cells and plasmablasts (**Supplementary Table 2**). For the three donors and two or three SHM bins, Tregitope content in plasmablasts was significantly lower than that of memory B cells (**Figure 4**; median score memory to plasmablasts ratio>1). JMX high content was also lower in plasmablasts but only for two donors and only for two SHM bins. Plasmablasts had higher and lower JMX low content than memory B cells (median score ratio<1 and ratio>1), and three out of four observations with significant differences were for donor 1. Compared to JMX low content, the significance level of lower Tregitope content in plasmablasts was consistently higher. Overall, these results showed that Tregitope content in memory B cell BCRs is higher than that of plasmablasts, which suggest that maturation to plasmablasts is associated with Tregitope loss during antibody development.

### 2.4 Tregitope loss occurs in multiple V-gene combinations and is the most frequent change in T cell epitope content

**Figure 5:**
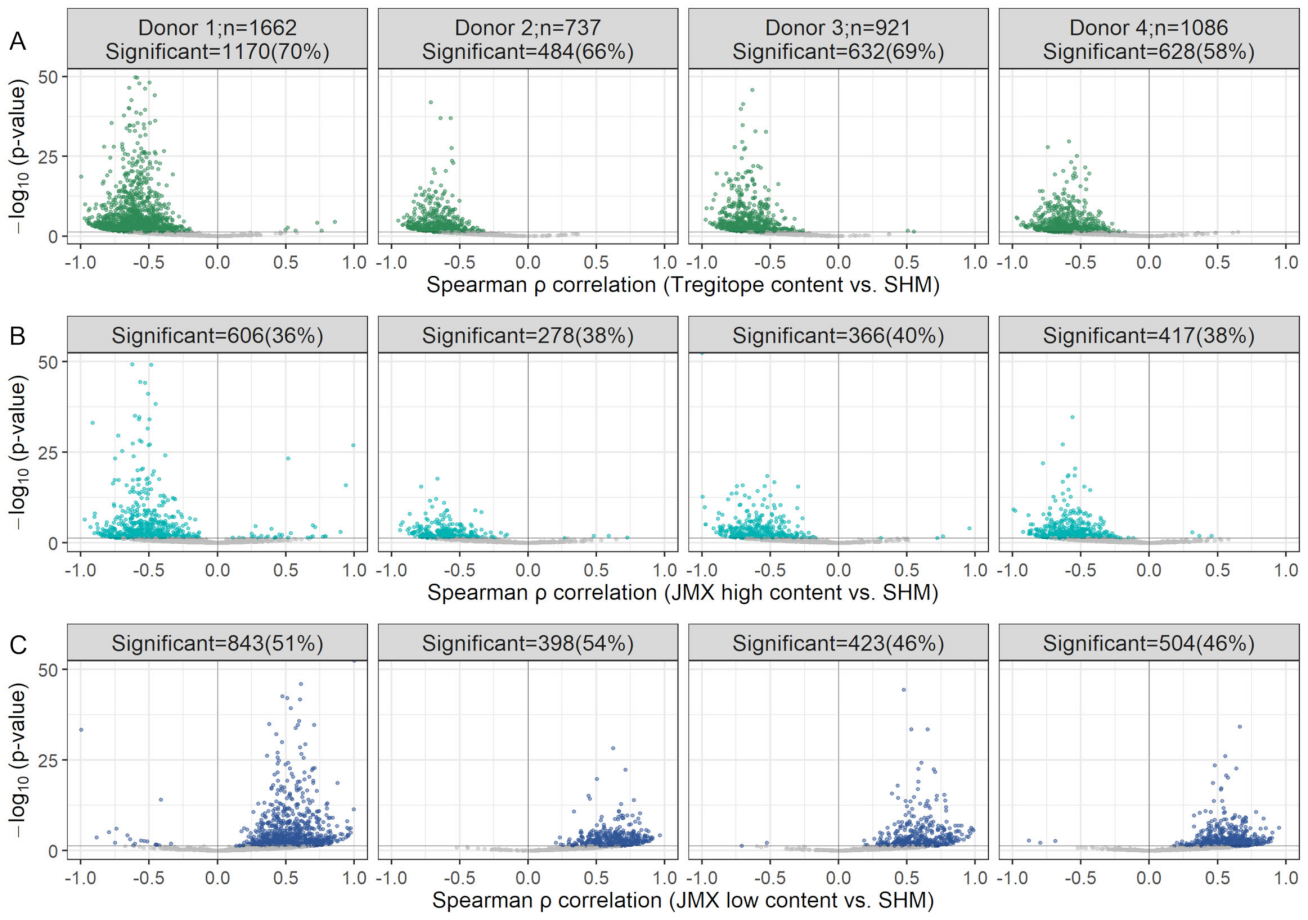
Decrease in Tregitope content and potentially tolerated T cell epitope content with SHM and increase in potential T effector content occur in several V-gene pair combinations. Volcano plots of Spearman ρ vs. Benjamini-Hochberg adjusted p-values for Tregitope **(A)**, JMX high **(B)**, and JMX low content **(C)** vs. SHM, for antibody repertoires grouped by IGHV and IGKV/IGLV gene pairs. Statistically significant V-gene pairs are colored; the remaining gene pairs are shown in gray. The number of pairs per donor is indicated in the top panel. The number of significant pairs per donor are indicated at the top of each plot. Y-axes were truncated to 50; pairs with p-values>50 were considered to calculate the number of significant pairs. Pairs with fewer than 10 antibodies were excluded.

Tregitope content, potentially tolerated T cell epitope content, and potential T effector content were correlated with SHM in the analysis of donor repertoires. To investigate whether any of these T cell epitope subsets of specific heavy and light chain V-gene pairs were correlated with SHM, antibodies were grouped by paired IGHV and IGKV/IGLV genes. Significant changes in each T cell epitope content subset were detectable in a multitude of V-gene combinations (**Figure 5**). More than 58%, 36%, and 46% of the V-gene pairs displayed significant correlations between SHM and Tregitope, JMX high, and JMX low content, respectively.

For Tregitope content and JMX high, 99.7% and 97.4% of the V-gene pairs with significant results, respectively showed loss of Tregitopes or potentially tolerated content with increasing SHM (i.e. negative Spearman ρ correlation). The remaining 0.3% and 2.6%, respectively, gain Tregitopes or potentially tolerated content. For JMX low, 98.9% of the V-gene pairs with significant results showed gain in potential T effector content with increasing SHM; the remaining 1.1% lost potential T effector content.

**Figure 6:**
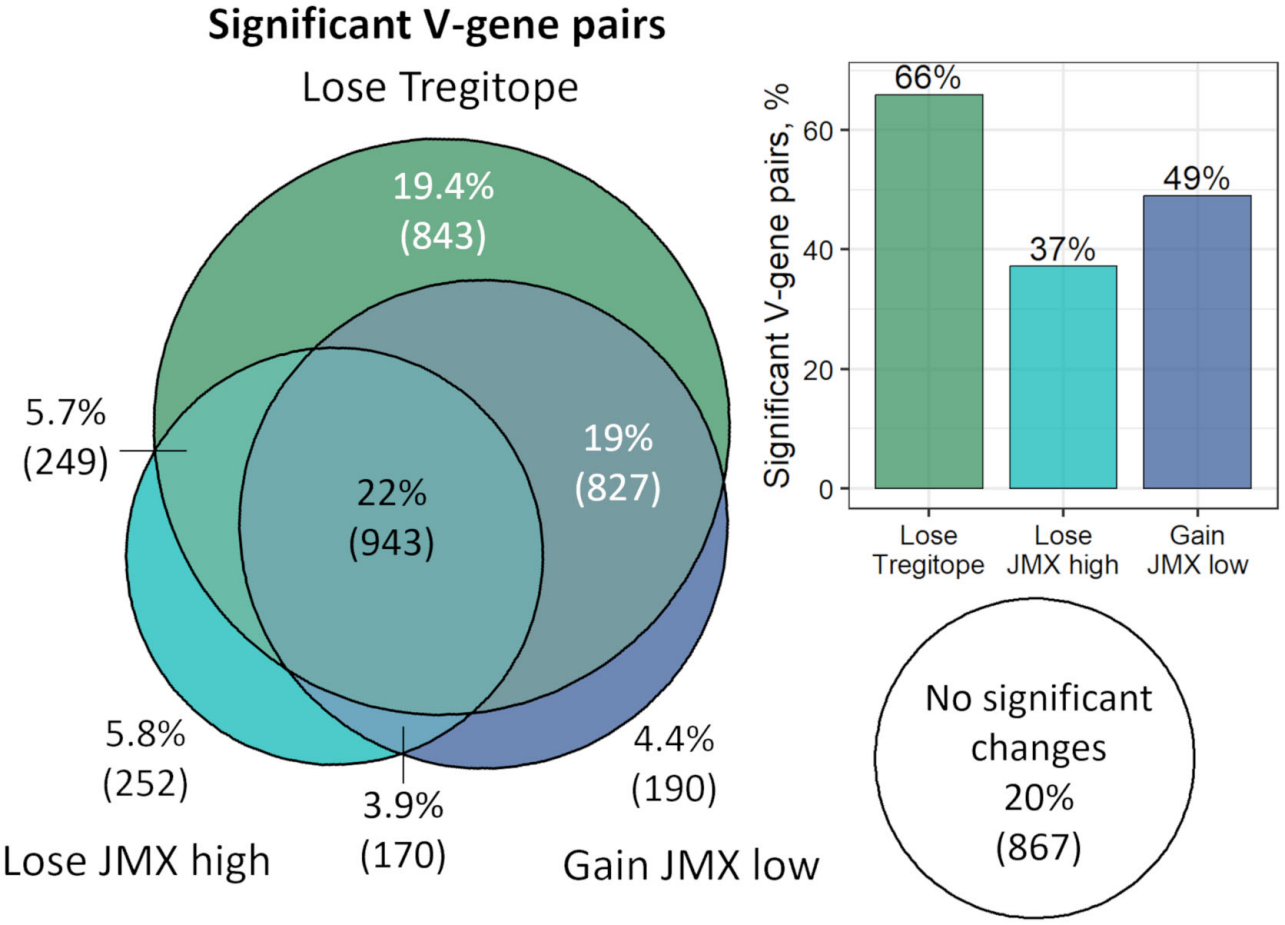
V-gene combinations lose Tregitope content more frequently than they lose potentially tolerated T cell epitopes or gain potential T effector epitopes. The Euler diagram shows the overlap of V-gene pairs across all four donors with significant correlation between T cell epitope content subsets and SHM. Percentage and number of V-gene pairs are shown. Areas are proportional to the number of pairs in each T cell epitope content subset. The bar plot shows the percentage of significant V-gene pairs for each T cell epitope content subset. Pairs that gained Tregitope (n=8) or JMX high (n=43) or lost JMX low (n=23) with SHM were excluded. Taking all the significant pairs that lose Tregitope or JMX high or gain JMX low content from the antibody repertoires from four donors, 22% were significant for Tregitope, JMX high and JMX low content. The largest overlap (19%) was for pairs that lose Tregitope content and gain potential T effector content (JMX low). A similar percentage (19.4%) of the V-gene pairs only lose Tregitope compared to the small percentages of pairs that only lose JMX high (5.8%) or only gain JMX low (4.4%). Overall, 66% of all the significant V-gene pairs lose Tregitope content, more than JMX high (37%) and JMX low (49%).

Several V-gene pairs had significant changes for more than one subset of T cell epitope content. Taking all the significant pairs that lose Tregitope or JMX high or gain JMX low content from the antibody repertoires from four donors, 22% were significant for Tregitope, JMX high and JMX low content (**Figure 6**). The largest overlap (19%) was for pairs that lose Tregitope content and gain potential T effector content (JMX low). A similar percentage (19.4%) of the V-gene pairs only lose Tregitope compared to the small percentages of pairs that only lose JMX high (5.8%) or only gain JMX low (4.4%). Overall, 66% of all the significant V-gene pairs lose Tregitope content, more than JMX high (37%) and JMX low (49%). These results demonstrated that V-gene combinations lose Tregitope content more frequently than they lose potentially tolerated T cell epitopes or gain potential T effector epitopes.

### 2.5 T cell epitope content changes in BCRs are driven by SHM mutational preferences

To evaluate whether changes in T cell epitope content subsets observed with increasing SHM were introduced as an indirect consequence of SHM mutational preferences or by selection pressure, we developed *in silico* simulations that accounted for donor-specific SHM DNA motif targets. The models did not account for HLA-DR-specific epitope selection pressure. Thus, stronger correlations between T cell epitope content subsets and SHM in donor repertoires than in donor-matched *in silico* models would indicate that epitope content changes driven by SHM (loss of Tregitopes, loss of potentially tolerated epitopes, or gain of potential T effector epitopes) were the result of active selection *in vivo*.

For each donor, we simulated 10 donor-specific antibody repertoires that matched the V-gene frequencies, 5-mer DNA targeting patterns, and SHM distribution in each individual donor repertoire (**Supplementary Figure 2**). The donor-specific in-frame replacement and silent mutations (RS) SHM model accounted for 5-mer nucleotide preferences of the human activation-induced cytidine deaminase (AID), the enzyme responsible for SHM, and biophysical restrictions on permissible DNA/amino acid mutations in functional BCRs.

For class-switched antibodies, comparisons between matched V-gene pairs (pairs that were significant for the donor and the model repertoires) showed that changes in T cell epitope content subsets with SHM were, in most cases, significantly different between donors’ repertoires and RS models; however, low rank-biserial correlation values suggest small effect sizes (**Supplementary Figure 3**), indicating that the differences between donor and modeled repertoires were not meaningful. Overall, these results suggest that *in silico* models that accounted for donor-specific V-gene usage and AID targeting preferences produced similar level of T cell epitope content change (Tregitope and JMX high loss and JMX low gain) observed in the donors’ repertoires, suggesting that these changes are a consequence of SHM mutational preferences.

### 2.6 Mutations introduced in identified Tregitopes by SHM alter HLA-DR binding affinity

The function of Tregitopes can be altered by reducing their HLA-DR binding affinity and/or modifying their TCR-facing residues. Just as for T effector epitopes, when HLA binding affinity is decreased, T cell responses may be compromised (reducing regulatory T cell activation). Thus, donor-specific modifications in residues that interact with HLA-DR and reduce binding or change the register of the epitope in the binding groove (34) may reduce Treg function. Mutations in TCR-facing residues of Tregitopes may also reduce Treg effect due to reduction in activation of their cognate Treg, rather than reduced binding.

To evaluate the effect of mutations introduced in Tregitopes by SHM, variants of Tregitopes from the VH region (Tregitopes 9A and 88) were selected for HLA binding assays based on predicted binding affinity and cross-conservation with human proteins. For each Tregitope, we selected two variants from the donors’ repertoires: one variant with reduced binding likelihood for a panel of seven alleles (HLA-DRB1*01:01, *03:01, *04:04, *11:01, *13:01, *15:01, and *16:01) that cover the alleles expressed by donors 1, 2, 3 and 4, and one variant with TCR-facing mutations that modified the potential for TCR recognition by their cognate T cell (JMX low; potential T effector epitope), even though they were still predicted to bind the donors’ HLA-DR alleles.

Compared to the germline sequences Tregitope 9A and 88, modified Tregitope 9A and 88 variants selected from the donors’ repertoires that were identified as having potentially reduced binding likelihood (**Table 1**; Treg9Av_rb and Treg88v_rb) did indeed not bind or had reduced HLA-DR binding affinity for a panel of five alleles tested (HLA-DRB1*01:01, *03:01, *04:01, *11:01, *15:01). These HLA-DR were selected for the assays because they represent the alleles expressed by donors 2, 3 and 4. The only exception to the expected outcome was an observed increase in the binding affinity of Tregitope 9A variant (Treg9Av_rb) for HLA-DRB1*04:01. Most variants with changes in the TCR-facing residues had similar or reduced binding affinity as compared to the original Tregitope sequences from which they were derived. These variants bind HLA-DR but may not activate cognate Tregs. Taken as a whole, these results showing decreased binding of selected Tregitope variants, or no change in binding in of selected TCR-facing Tregitope variants, are consistent with our expectations.

**Table 1:** HLA-DR binding of Tregitopes and variants with reduced binding likelihood and human cross-conservation.

Label	Version	Selection criteria	Expected HLA-DR binding	Expected Treg function	Sequence^a^	EMX hits^b^	JMX score^b^	HLA-DRB1 (IC_50_ nM)				
								*01:01 (d2)	*03:01 (d4)	*04:01 (d3)^c^	*11:01 (d3)	*15:01 (d2,d4)
Treg9A	Original	Tregitope	Promiscuous	Normal	GGLVQPGGSLRLSCAASGFTF	11	27.64	704	819	3,222	221	289
Treg9Av_rb	Variant	Reduced binding likelihood; EMX low	Reduced	Reduced; decreased HLA binding affinity	GGL**A**QPGGSLRLSC**EV**S**NV**T**S**	0	0.00	non-binder	non-binder	896	non-binder	22,918
Treg9Av_rxc	Variant	Reduced human cross-conservation; JMX low	Promiscuous	Reduced; decreased cognate Treg activation	GGL**A**QPGGSLRLSC**TP**SGF**I**F	7	0.14	non-binder	61,559	125	43,530	4,612
Treg88	Original	Tregitope	Promiscuous	Normal	NTLYLQMNSLRAEDTA	15	22.40	200	2,661	112	299	2,140
Treg88v_rb	Variant	Reduced binding likelihood; EMX low	Reduced	Reduced; decreased HLA binding affinity	NT**AS**L**H**M**HD**LR**P**ED**SG**	0	0.00	non-binder	non-binder	non-binder	non-binder	non-binder
Treg88v_rxc	Variant	Reduced human cross-conservation; JMX low	Promiscuous	Reduced; decreased cognate Treg activation	N**S**L**F**L**H**M**DN**LRAED**S**A	10	0.00	168	7,731	85	755	4,469

a Amino acid differences compared to original Tregitope sequences are shown in red font.

b EpiMatrix (EMX) hits and JanusMatrix (JMX) score calculated for all the HLA-DR alleles expressed by donors 2, 3, and 4 (d2, d3, and d4) (HLA-DRB1*01:01, *03:01, *04:04, *11:01, *13:01, *15:01, and *16:01).

c HLA-DRB1*04:01 used instead of *04:04 (expressed by donor 3). These alleles share similar binding peptide side-chain preferences for binding pockets.

Non-binder (IC_50_ >100,000nM).

Low affinity (IC_50_ 10,000-100,000nM).

Moderate affinity (IC_50_ 1,000-10,000nM).

High affinity (IC_50_ 100-1,000nM).

Very high affinity (IC_50_ <100nM).

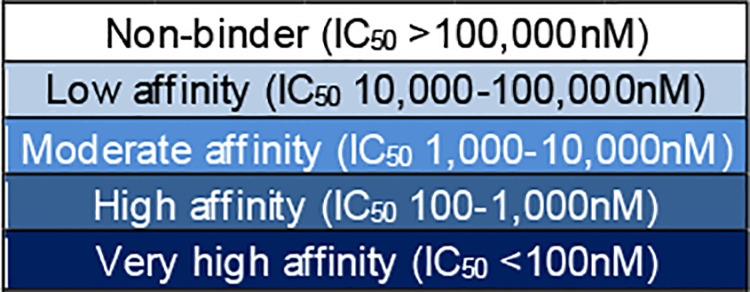

### 2.7 Tregitopes removed during SHM inhibit proliferation of CD4+ T cells

Validation of Tregitopes can be performed using a wide range of assays, including T cell phenotype and *in vitro* or *in vivo* effect on T effector or inflammatory immune responses. We developed a bystander suppression assay that demonstrates the effect of Tregitopes *in vitro* when Tregitope peptides are present in the same well with a stimulatory antigen such as tetanus toxoid (TT) (35). After demonstrating their promiscuous HLA-DR binding, we confirmed the regulatory effect of Tregitopes 9A and 88 for which inhibitory data were not previously published, by measuring their inhibition of the recall response of human CD4+ T cells to the TT antigen in the standardized Bystander Suppression Assay (TTBSA), using peripheral blood mononuclear cells (PBMCs) from donors possessing some of the HLA-DR alleles to those from which antibody repertoires were isolated. In previous studies, we have performed the same assay with other Tregitopes (35).

**Figure 7:**
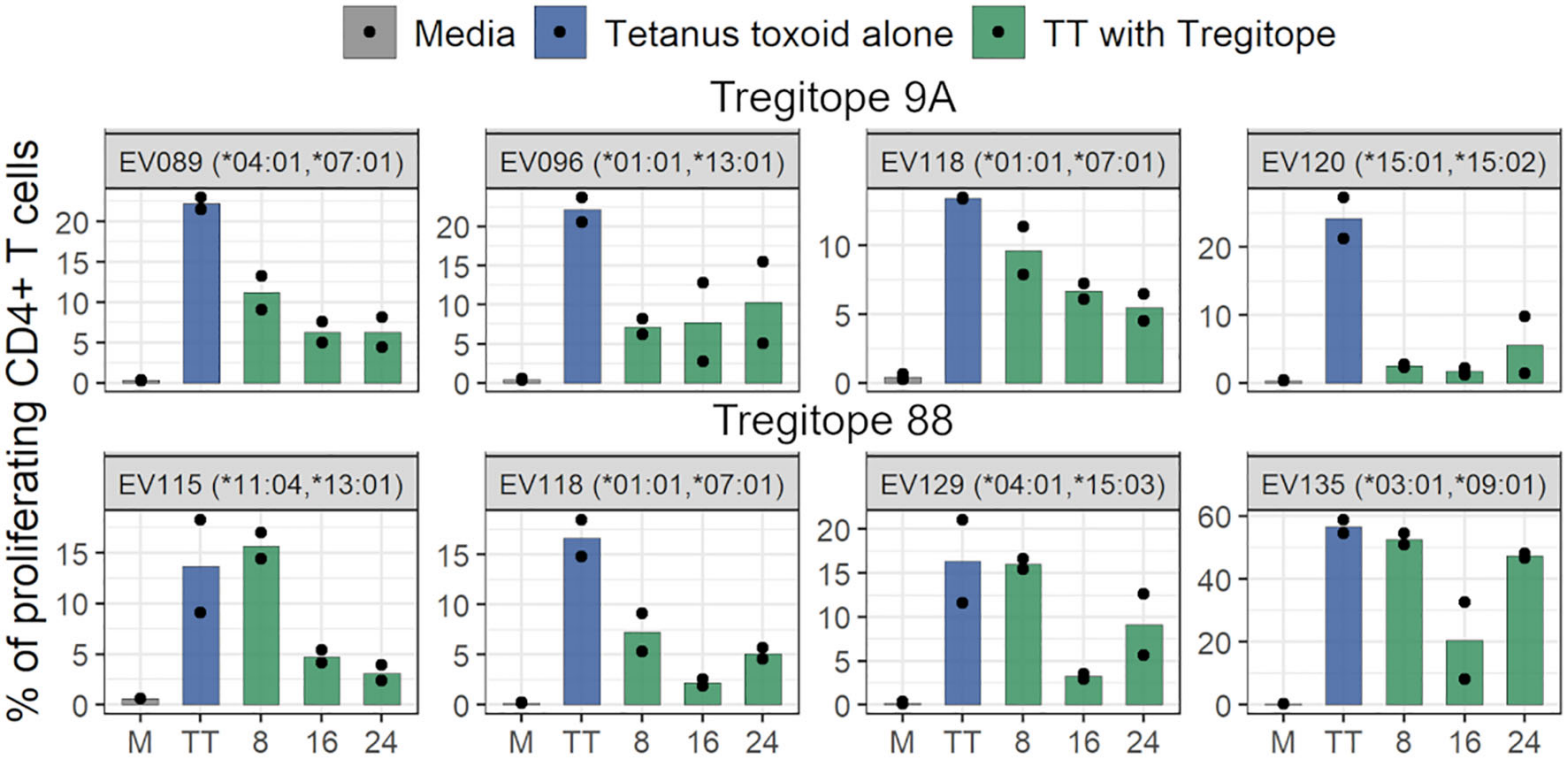
Tregitopes inhibit proliferation of CD4 T cells. The bar plots show the inhibition of CD4+ T cell recall response by Tregitopes 9A and 88 in the TTBSA. PBMCs from healthy donors were stimulated with 0.5 μg/mL of TT with or without the addition of the indicated concentrations of Tregitopes and analyzed at day six post-stimulation by flow cytometry. The effect of Tregitope co-stimulation with TT on the inhibition of CD4+ T cell proliferation by Tregitopes 9A and 88 were plotted.

Tregitope peptides 9A and 88, that had been identified as being among the Tregitopes removed in this study, were tested at three different concentrations (8, 16, and 24 μg/mL). TT stimulated proliferation of CD4+ T cells is shown at the left (in blue) in each graph in **Figure 7**. Stimulation with Tregitope 9A and 88, along with TT *in vitro*, suppressed recall CD4+ T cell responses specific to the TT antigen.

## 3 Discussion

Our group has identified several regulatory T cell epitopes (Tregitopes) derived from conserved Fc and Fab sequences of IgG and located in antibody VH and VL framework regions or framework/CDR regions. These Tregitopes have been validated in our own laboratory and were recently re-identified as tolerogenic, tTreg activating T cell epitopes (23). To investigate whether Tregitopes that may be recognized by Tfr are removed from BCRs during antibody maturation, we performed an *in silico* analysis of the dynamics of donor-specific T cell epitope content in the repertoire of natively paired antibody VH and VL chains from donors with known HLA-DR alleles studied by Jaffe et al. (27). The results showed that SHM leads to Tregitope depletion in BCRs and that Tregitope depletion is positively associated with class-switching, and maturation to plasmablasts. These findings reveal a new mechanism of regulation of antibody maturation.

In previous work, our group has determined that the presence or absence of Tregitope sequences within the variable domains of monoclonal antibodies and other antibody-like (36) molecules is a useful predictor of immunogenic potential (the induction of ADA) (25, 26). In addition, we have shown that when delivered systemically, Tregitope peptides can suppress inflammatory immune response (37). And, that when co-delivered with antigen, Tregitope peptides can induce antigen-specific immune tolerance (38–40). Tregitopes may be applied anywhere active immune response is activated, including in the treatment of autoimmune disease (41), in the treatment of inflammatory conditions (42), in the suppression of transplant rejection (37), and to induce tolerance to therapeutic proteins including monoclonal antibodies, “replacement” proteins such as FVIII (35), and alglucosidase alfa (Myozyme) (43).

Despite these many illustrations of their utility, the natural function of Tregitopes has not been uncovered, to date. In this report, we show that Tregitope content decreases as SHM increases, implicating a natural role for Tregitopes in the process of antibody maturation. Previously, we believed that the natural function of Tregitopes was to suppress anti-idiotype immune response thereby helping to maintain immune homeostasis. While this may still be true and is relevant to the maintenance of homeostasis in the B cell follicle in the absence of antigenic stimulation, the depletion of Tregitopes appears to be also associated with an increase in potential T cell epitope content in the antibodies that are developing during SHM.

**Figure 8:**
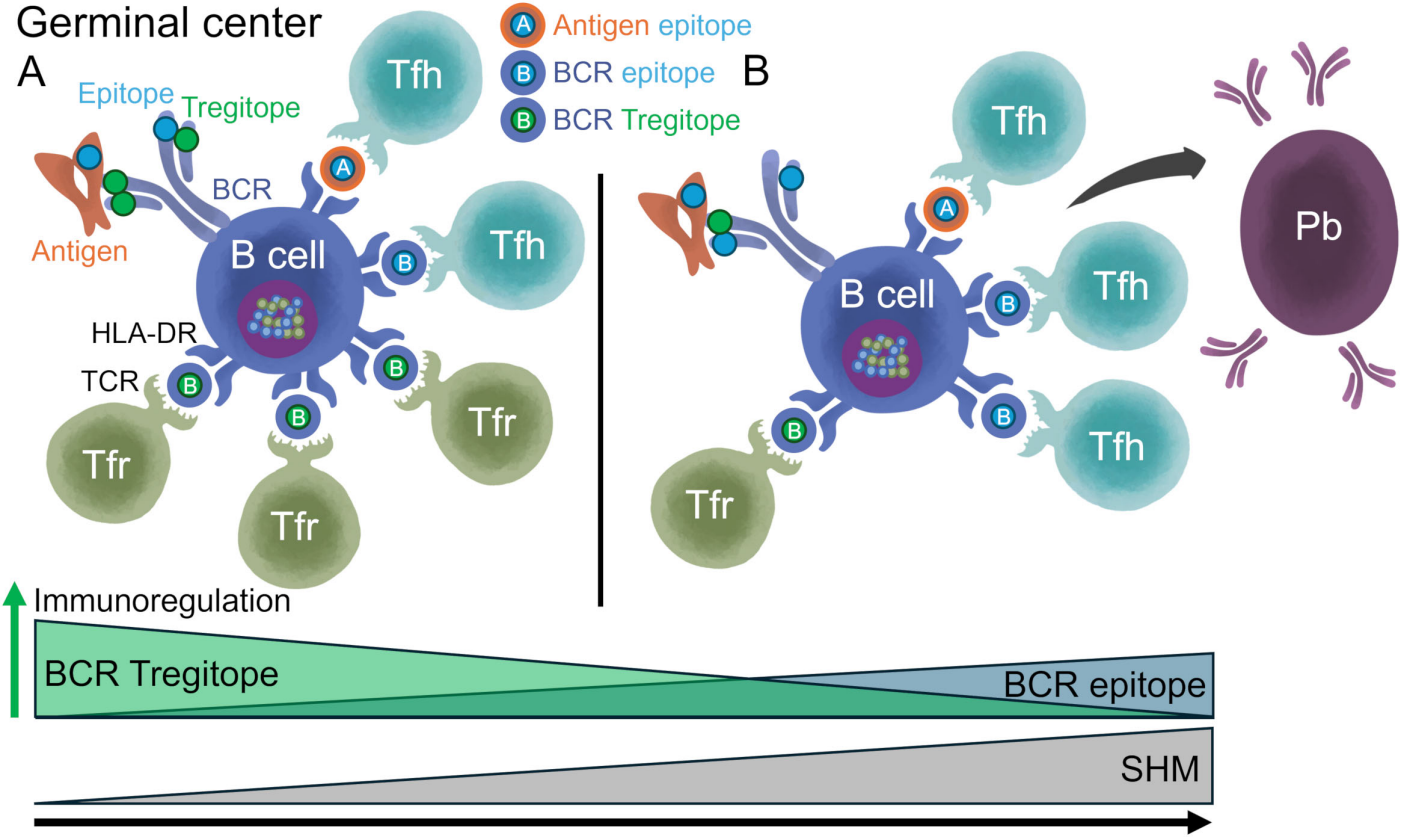
Model of *in vivo* selection of antibodies with decreased Tregitope content and increased T effector content. **(A)** GC B cells capture foreign antigens via BCR-mediated endocytosis; antigen and BCR peptides that bind HLA-DR are transported to the surface of the cell for presentation to Tfh and Tfr. Unmutated BCRs contain high Tregitope content. Tregitopes activate Tfr, which regulate B cell metabolism, GC formation, and autoreactive B cells activation. In the absence of an antigen, Tregitope:HLA-DR:Tfr interactions may play a role in maintaining immune homeostasis. **(B)** BCRs with decreased Tregitope and potentially tolerated content and increased potential T effector epitope content are selected in the GC leading to decreased activation of Tfr cells, increased SHM, formation of antigen-specific Tfh cells, and GCs. Tfr cells still suppress autoreactive B cells. Low Tregitope content in BCRs is associated with class-switching and maturation to plasmablasts (Pb). Once antibodies are in circulation, an anti-idiotypic immune response may develop to clear immunogenic antibodies when the antigenic load diminishes. We note that this analysis did demonstrate donor-specific HLA-DR T cell epitope removal. This occurred even though SHM does not specifically target HLA-specific binding amino acids. Instead, changes to the antibody sequence that result in reduced HLA-DR binding (or reduced TCR engagement by Tregs) are likely to enable B cells that carry those modified sequence (as in **B**) to escape immune response (i.e., positively selected in the GC).

T cell-mediated affinity maturation, class-switching, and maturation to plasmablasts appear to be correlated with the loss of Tregitope content suggesting an important role in the development of durable antibody immune responses. We observed significant loss of Tregitope content in all antibody isotypes (IgG, IgA, and IgM). However, class-switched IgA and IgG antibodies had greater correlation of Tregitope loss with SHM and lower Tregitope content than IgM, suggesting an association between Tregitope removal and class-switching mediated by T cell responses. In addition, our analysis of the antibody repertoire grouped by paired antibody heavy and light chain V-genes showed that the loss of Tregitope content with SHM occurs in multiple combinations of V-gene pairs and is more frequent than loss of potentially tolerated or gain of potential T effector content. We also observed that class-switched antibody sequences of plasmablasts had significantly lower Tregitope content than those of memory B cells, which suggest that B cells with low Tregitope content BCRs may be selected in the GCs for differentiation and maturation (**Figure 8**).

Comparing donors’ repertoires to large-scale *in silico* simulated antibody repertoires that accounted for donor-specific SHM DNA motif targets but not for HLA-DR-specific epitope selection pressure, we found that changes in T cell epitope content are likely a consequence of SHM mutational preferences. While differences in T cell epitope content change with SHM between donors’ repertoires and simulated repertoires were significant, they were not meaningful considering their effect sizes. Using a similar approach to simulate antibody repertoires, Gutiérrez-González M. et al. showed that SHM selectively targeted donor-specific HLA-DR T cell epitopes (20). Differences in the algorithms used for prediction of T cell epitopes, the number of simulated repertoires, and the metrics applied to define T cell epitope content change with SHM, may explain differences in the results. Despite these differences, both studies demonstrated donor-specific HLA-DR T cell epitope removal in antibody variable regions in repertoires from nine different donors. Furthermore, we found that the overall loss of T cell epitope content with SHM is mainly driven by Tregitope depletion, with a significant contribution from potentially tolerated T cell epitopes. In contrast, potential T effector epitope content increased with SHM.

Similar to previously reported Tregitopes (35), Tregitopes 9A and 88 (which were among the Tregitopes depleted in donor repertoires) inhibited antigen-specific CD4+ T cell proliferation. Amino acid modifications in Tregitopes introduced by SHM may impact Treg function by reducing or eliminating HLA-DR binding as well as cognate Treg TCR interaction and activation. To evaluate the effect of such modifications in Tregitopes 9A and 88, we identified two variants with low binding likelihood and two variants with modified TCR-facing residues. The variants with low binding potential had reduced HLA-DR binding affinity *in vitro*. Variants with TCR-facing modifications, which may reduce cognate Treg activation, had similar or reduced binding affinity when compared to the original Tregitopes. It is possible that changes in the TCR-facing residues affected HLA binding. While these HLA-DR specific modifications seem to be driven by SHM mutational preferences, they might “protect” the B cell bearing the modified BCR from the restraining influence of Tfr in the GC. We note that while this analysis of B cell repertoires did demonstrate donor-specific HLA-DR T cell epitope removal, SHM does not specifically target HLA-specific binding amino acids. Instead, changes to the antibody sequence that result in reduced HLA binding (or reduced TCR engagement by Tregs) appear to be more likely to enable B cells that carry those modified sequence (as in **Figure 8B**) to escape immune response (i.e., positively selected in the GC).

Treg cells in the B cell follicle regulate GC response by modulating activity of both Tfh cells and B cells. Tfr are less abundant than Tfh cells in the GCs and are also less frequently found in the GCs than in the B cell follicle (44, 45). However, Tfr cells have a modulatory role controlling initial GC formation (15). In the GC, B cell-Tfr interactions facilitated by chemotactic targeting, control B cell selection (46, 47). The removal of Tregitopes (and potentially tolerated T cell epitopes) in maturing antibodies may serve to decrease the activation of Tfr cells leading to increased formation of antigen-specific Tfh cells, GC, memory B cells, and antibody-secreting cells (**Figure 8**). Thus, the role Tregitopes may play is to maintain immune homeostasis when the antibodies in which they are found are not being “applied” to immune response. Once immune response begins in the B cell follicle, the removal of Tregitopes through SHM “releases the brake” on B cells. BCRs with lower Tregitope content are selected for clonal expansion, allowing for the development of a more robust antigen-specific immune response.

This theory is supported by the literature. In models of viral infection and immunization, Tfr controlled SHM and clonal diversity by restraining non-antigen-specific GC B cells and limiting clonal competition, which promoted affinity maturation of antigen-specific B cells (29). Limiting Tfh cells reduced SHM, antigen-specific GC responses, affinity maturation, and clonal diversity (29). The coincident addition of new T effector epitopes in maturing GC B cells could enhance Tfh responses, supporting the importance of immunogenicity in the development of antigen-specific immune response. Overall, antibodies that have higher SHM appear to have more immunogenic potential themselves, suggesting that an anti-idiotypic immune response may develop once they are in circulation, which could lead to clearance of these high affinity antibodies when the antigenic load diminishes, supporting Jerne’s idiotype-anti-idiotype interaction hypothesis (30).

While the removal of Treg epitopes to improve the maturation of antibodies makes sense teleologically, the enzyme that modifies antibodies (AID) does not, to our knowledge, target any specific epitope. Nonetheless, modification of an epitope sequence may have different outcomes for Tregitopes and potentially tolerated epitopes (JMX high) than for potential T effector epitopes (JMX low). In the case of the former, a modification to the TCR-facing region could reduce human homology, and therefore no longer be counted as potentially tolerogenic (the epitope moves from Treg or tolerated to T effector). In the case of a T effector epitope, a modification that does not introduce cross-conservation with the human proteome would be counted as potentially immunogenic (from T effector to T effector). Thus, the impact of modifications is different for these two categories of T cell epitopes.

Our findings have implications for designers of monoclonal antibody and antibody-like molecules. As mentioned above, we have shown a significant correlation between clinical immunogenicity (as measured by ADA) and lack of Tregitope content in monoclonal antibodies (25, 26). Where antibodies are intended for use in immune competent subjects, use of Tregitope replete germlines as a scaffold for antibody development is generally preferred. However, studies of immunogenicity in the context of immunomodulatory drugs such as checkpoint inhibitors, have suggested the use of Tregitope replete germlines may be counterproductive, leading to reduced efficacy (48). Furthermore, we have hypothesized that where the regulatory T cell compartment of intended patient populations is compromised (as in, Tregs are not functioning as they usually do), the use of Tregitope replete germlines may contribute to the activation of effector T cells and enhanced ADA response.

In the future, we hope to determine whether the SHM is impaired in patients who have poorly functioning Tfr cells, which would support our hypothesis that Tfr cells activated by Tregitopes put a “brake” on SHM. Studies in an immunization model showed that Tfr deficiency led to reduced SHM and increase in non-vaccine-specific GC B cells (29). In patients with autoimmunity, self-reactive antibodies routinely escape from immune modulation (potentially due to the lack of effective regulatory T cell responses in the B cell follicle) and “self” antigens are targeted (49).

Further studies may be able to elucidate how Tregitope-specific Tregs modify immune response to antibodies in the lymphoid follicle. Treg cells can deplete peptide-MHC-II complex from dendritic cells through trogocytosis to limit antigen presentation and priming of naïve antigen-specific T cells (50). Tregs can also deplete CD80 and CD86 molecules on dendritic cells in a similar manner (51). We have demonstrated a decrease in HLA-DR expression in dendritic cells co-incubated with Tregitopes and Treg cells, as well as downregulation of CD80 and CD86 (35). Studies in mice showed that Tfr TCR repertoire was distinct from that of Tfh and it closely resembled the thymic regulatory Treg repertoire, suggesting that the Tfr TCR repertoire overlaps with tTregs and its antigen specificity is directed towards self-antigens (46, 52, 53). If Tfr cells specificity is for self-epitopes and Tregitopes, and distinct from Tfh cells specificity, Tfr may regulate (or deplete) GC B cells with BCRs that contain high Tregitope content, enhancing immune response and SHM. With reduced Tregitope presentation, GC B cells gain in the competition as compared to other GC B cells presenting more Tregitopes, improving their ability to elicit Tfh cell help (**Figure 8**). This would be another level of control to stimulate the development of Tregitope-depleted BCR-bearing B cells and to inhibit the outgrowth of autoreactive or non-antigen specific B cell clones.

In summary, we found a significant correlation between donor-specific Tregitope depletion and molecular markers of antibody maturation mediated by T cells. These data reveal a novel regulation mechanism of human antibody maturation that is relevant to immune responses for vaccines and pathogens, to the lack of regulation of autoantibodies, and for the selection of therapeutic antibody candidates.

## 4 Materials and methods

### 4.1 Antibody sequences

#### 4.1.1 Donor repertoires

Previously published antibody repertoires from four human subjects with known HLA-DR alleles were analyzed (27). Using PBMCs, single B cells were captured, and natively paired antibody heavy and light chains were sequenced. B cell phenotypes were defined by flow cytometry (27). The following flow gating definitions were used for memory and plasmablast populations: unswitched memory: live, CD3^−^CD19^+^CD27^+^IgD^low^IgM^++^CD38 ^±^ CD24^±^; switched memory: live, CD3^−^CD19^+^CD27^+^IgD^−^CD38 ^±^ CD24 ^±^ CD95^±^; and plasmablast: live, CD3^−^CD19^+^CD27^+^IgD^−^CD38^++^CD24^−^. IgBLAST was used for V(D)J annotation (54). SHM percentages were determined based on the identify percentage between heavy and light chain V-genes and their corresponding germlines. Isotype assignment was performed by matching constant region sequences to isotype barcodes (27). Amino acid sequences of BCRs of memory B cells and plasmablasts were extracted from the IgBLAST output for HLA-DR T cell epitope prediction using EpiMatrix (26). Thus, each antibody repertoire was comprised of natively paired antibody heavy and light amino acid sequences, their isotypes, and IgBLAST annotation (VH and VL SHM percentages, IGVH and IGVL genes). Only antibodies with full-length VH and VL sequences were analyzed.

#### 4.1.2 Modeled repertoires

Donor-specific *in silico* models were developed using immuneSIM for V(D)J recombination modeling (55) and ShaZam for SHM modeling (56) as previously described (20). Briefly, to generate naïve repertoires, the distribution of V-genes of memory B cells for each donor repertoire was calculated and used to build frequency tables, which were used as custom vdj_list parameter in the immuneSIM function. Naïve repertoires for heavy and light chains were generated separately. To simulate SHM, donor-specific replacement-silent SHM targeting models were generated using the createTargetingModel function from ShaZam. Using this personalized models, sequences from the naïve repertoire were mutated individually with the shmulateSeq function from ShaZam. For each donor, the number of mutations per sequence was set to match individual SHM distribution. Next, each modeled repertoire was annotated using IgBLAST. Productive variable heavy and light sequences were then paired following the SHM distribution of each donor repertoire. V-gene and SHM distributions of each model were compared to the donor repertoire to confirm their similarity. Amino acid sequences were extracted from the IgBLAST output for HLA-DR T cell epitope prediction using EpiMatrix. Ten RS models of the same size of each donor repertoire were generated, for a total of 2,102,680 antibodies. Isotypes were assigned following the donor distribution.

### 4.2 Prediction and analysis of personalized T cell epitope content

The pipeline used for prediction and analysis of personalized T cell epitope content is shown in **Supplementary Figure 4**. The antibody sequences were scored using EpiVax’s proprietary algorithms available in ISPRI. The EpiMatrix algorithm was used to screen paired VH and VL antibody sequences for potential donor-specific HLA-DR T cell epitopes (26). Each sequence was parsed into overlapping 9-mer frames and assessed for their binding potential to the donor’s HLA-DR alleles. EpiMatrix frame-by-allele assessment Z-scores range from approximately -3 to +3 and are normally distributed. EpiMatrix Z-scores above 1.64 are considered potential HLA binders. T cell epitope prediction was performed for donor-specific HLA-DR alleles: HLA-DRB1*13:01, 16:01 (donor 1), 01:01, 15:01 (donor 2), 04:04, 11:01 (donor 3), and 03:01, 15:01 (donor 4).

Predicted T cell epitopes were further classified as known Tregitopes, JanusMatrix high (JMX high) or JanusMatrix low (JMX low) based on their cross-conservation with human epitopes predicted using JanusMatrix (31). T cell epitopes cross-conserved with three or more putative human T cell epitope 9-mers were categorized as JMX high and considered potentially tolerated. T cell epitopes cross-conserved with fewer than three putative human T cell epitope 9-mers were categorized as JMX low and considered potential T effector epitopes.

For each antibody, T cell epitope content was calculated by summing the EpiMatrix Z-scores of T cell epitopes predicted to bind the individual donor’s HLA-DR alleles. Tregitope content, JMX high content, and JMX low content were calculated by summing the EpiMatrix Z-scores of T cell epitopes in the corresponding subset. T cell epitope, Tregitope, JMX high, and JMX low contents were calculated for donor repertoires.

For the donor repertories, the relationship between SHM and each T cell epitope content subset was evaluated using Spearman ρ correlations and linear models fitted to calculate slopes and determine y-intercepts. Spearman ρ correlations were also used to evaluate the relationship between SHM each T cell epitope content subset in antibodies grouped by isotype. Spearman ρ correlation coefficients were compared between isotypes using a Wilcoxon rank sum test.

To compare the T cell epitope content for antibodies with similar SHM by isotype, SHM was truncated to 15% to capture more than 70% of the antibodies per donor. Antibodies were binned based on SHM using a 5% range. Comparisons between isotypes were performed using a Wilcoxon rank sum test.

To compare the T cell epitope content of isotype-switched antibody sequences of memory B cells and plasmablasts, SHM was truncated to 20% to capture more than 70% of the antibodies per donor. Antibodies were binned based on SHM using a 5% range. T cell epitope content subsets of B cells and plasmablasts were compared using a Wilcoxon rank sum test. The ratio of the medians between memory B cells and plasmablasts for each T cell content subset was calculated for each bin. BCR sequences of plasmablasts were available for donors 1, 3, and 4. Only this analysis included plasmablasts. For all the other analyses, donor repertoires included BCRs from memory B cells.

### 4.3 In-depth analysis of T cell epitope content dynamics

To evaluate the relationship between the T cell epitope content subsets of specific heavy and light chain V-gene pairs with SHM, BCRs from memory B cells were grouped by paired IGHV and IGKV/IGLV genes. The statistical significance for each V-gene pair was determined by calculating the Spearman correlation with p-values adjusted for multiple comparisons using the Benjamini-Hochberg method. Groups of V-gene pairs with 10 or more antibodies were included. The Spearman ρ correlations and p-values of paired V-genes groups that showed significant correlation between each T cell epitope content subset and SHM, were compared using Wilcoxon rank sum tests.

### 4.4 Tregitope variant selection

Variants of Tregitope 9A and 88 with reduced binding likelihood or reduced cross-conservation with human proteins (potential T effector epitopes) were identified to evaluate the effect of mutations introduced by SHM on Tregitope function *in vitro*. Tregitope variants from the donors’ repertoires, donor-specific EpiMatrix, and JanusMatrix scores were compiled and compared to Tregitopes. Variants with lower EpiMatrix score and JanusMatrix scores for the donors’ HLA-DR alleles (HLA-DRB1*01:01, *03:01, *04:04, *11:01, *13:01, *15:01, and *16:01) were identified. For each Tregitope, one variant with reduced binding likelihood (low EpiMatrix score) and one variant with reduced cross-conservation with human proteins (low JanusMatrix score) were selected for peptide synthesis.

### 4.5 Peptide synthesis

Six peptides (Tregitopes 9A, 88, two variants with reduced binding likelihood and two variants with reduced cross-conservation with human proteins) were synthesized by CPC Scientific Inc. (San Jose, CA). Molecular weight accuracy was verified by mass spectrometry. Using HPLC, peptides were more than 95% pure. To normalize net peptide content across assays, amino acid analysis was performed on all peptide samples.

### 4.6 HLA binding assays

HLA binding assays were performed as previously described (57). The alleles used for the binding assays were HLA-DRB1*01:01, *03:01, *04:01, *11:01, and *15:01, which covered the alleles expressed by donors 2, 3 and 4. For donor 3, HLA-DRB1*04:01 was used instead of HLA-DRB1*04:04; both alleles share similar binding peptide side-chain preferences for binding pockets. HLA-DRB1*13:01 and *16:01 alleles, expressed by donor 1 were not available for binding assays.

### 4.7 Tetanus toxoid bystander suppression assay

The regulatory effect of Tregitopes was evaluated using the tetanus toxoid bystander suppression assay (TTBSA) as previously described (35). Briefly, PBMCs from anonymous blood bank blood donors were labeled with CFSE cell proliferation dye and then plated at 3 × 10^5^ cells per well on U-bottom 96-wells plates in RPMI complete media. Labeled cells were rested overnight at 37°C, 5% CO_2_. The following day, the cultures were stimulated with Tregitopes 9A and 88. Peptides were solubilized in DMSO and added to the culture medium at a range of concentrations (8, 16, 24 μg/mL). Tetanus toxoid (0.5 μg/mL) was added to all wells including positive control wells without Tregitopes. The cells were cultured for 6 days, harvested at day seven, and stained for expression of cell surface and intracellular markers and analyzed by flow cytometry. Staining and gating was performed as previously described (35). Stained cells were acquired, and data collected was analyzed using FlowJo software (Treestar, Inc). Proliferation of CD4+ T cells was evaluated by dilution of CFSE with the proliferating population identified as CFSE_low_.

### 4.8 Statistical analysis

Statistical analyses were performed using R. For multiple comparisons, p-values were adjusted using the Benjamini-Hochberg method from the stats package. All correlations were calculated using the Spearman method from the stats package. Differences in the Spearman ρ correlation between isotypes were evaluated using a Wilcoxon rank sum test from the stats package. Differences in the Spearman ρ correlation and p-values of paired V-gene groups that showed significant correlation between each T cell epitope content subset and SHM were evaluated using a Wilcoxon rank sum test. The same test was applied for comparisons by isotype using binned SHM and between memory B cells and plasmablasts. For comparisons between donor and modeled repertoires, differences in the Spearman ρ correlation for IGVH and IGVK/IGVL gene pairs were evaluated using a Wilcoxon rank sum test. P-values below 0.05 were considered statistically significant. Effect sizes were defined using rank-biserial correlations from the effectsize package. Rank-biserial correlation is applied for non-parametric tests that use paired samples.

## Data Availability

The antibody repertoires were published previously (27). All of the analysis performed by the authors is included in the article and **Supplementary Material**. Access to the ISPRI platform is provided to commercial customers for a fee, and guided access is available at no cost to non-commercial academic researchers upon request and submission of a clearly outlined research proposal. Further inquiries can be directed to Anne S. De Groot (AnnieD@EpiVax.com).

## Supplementary material

Supplementary Figure 1 Comparison of Tregitope content among isotypes for antibodies with similar SHM. Box plots show Tregitope content in antibodies grouped by isotype and SHM. SHM was truncated to 15% and antibodies were binned based on SHM (5% range; bins: 0-5, 5-10, 10-15). Tregitope content was compared within each SHM bin using a Wilcoxon rank sum test. The median Tregitope content per isotype is shown at the bottom of each panel. Statistical significance is shown as *: p ≤ 0.05, **: p ≤ 0.01, ***: p ≤ 0.001, ****: p ≤ 0.0001.

Supplementary Figure 2 Comparison between donor and modeled repertoires. **(A)** VH gene usage (frequency) and **(B)** SHM distribution comparison between donor and modeled repertoire using donor-specific replacement-silent (RS) SHM models. We simulated 10 donor-specific antibody repertoires that matched the V-gene frequencies, 5-mer DNA targeting patterns, and SHM distribution in each individual donor repertoire.

Supplementary Figure 3 Comparison of T cell epitope content changes with SHM between donor and modeled class-switched repertoires. The Spearman p correlation coefficients of V-gene pairs with significant correlation between T cell epitope content subsets and SHM for the donor and replacement-silent (RS) switched modeled repertoires were compared using paired samples Wilcoxon rank sum test. V-gene pairs that were significant for both the donor and the model repertoires were included in the comparison. For each comparison, p-values, the cumulative number of compared pairs between donor repertoires and each of the 10 RS models and rank-biserial coefficients (rb) are shown. Effect sizes were defined using rank-biserial correlations; values range from -1, indicating complete dominance of the model sample (all the Spearman p correlation coefficients of the model are larger than all the coefficients of the donor), to +1, indicating complete dominance of the donor sample (all the coefficients of the donor are larger than all the coefficients of the model). Negative rb for Tregitope and JMX high indicates that a larger proportion of V-gene pairs from the donors’ repertoires had lower (negative) Spearman correlation coefficients compared to their modeled repertoires. Positive rb for JMX low indicates that a larger proportion of V-gene pairs from the donors’ repertoires had higher (positive) Spearman correlation coefficients compared to their modeled repertoires.

Supplementary Figure 4 Prediction and analysis pipeline of personalized T cell epitope content.

Supplementary Table 1 Spearman ρ correlation between T cell epitope content subsets and SHM by isotype.

Supplementary Table 2 Comparison of T cell epitope content subsets between switched memory B cells and plasmablasts (Pb).

## References

[1] Schatz DG Oettinger MA Baltimore D. The V(D)J recombination activating gene, RAG-1. Cell. (1989) 59:1035–48. doi: 10.1016/0092-8674(89)90760-5 2598259

[2] Briney B Inderbitzin A Joyce C Burton DR. Commonality despite exceptional diversity in the baseline human antibody repertoire. Nature. (2019) 566:393–7. doi: 10.1038/s41586-019-0879-y PMC6411386 30664748

[3] Rajewsky K. Clonal selection and learning in the antibody system. Nature. (1996) 381:751–8. doi: 10.1038/381751a0 8657279

[4] Cyster JG Allen CDC. B cell responses: cell interaction dynamics and decisions. Cell. (2019) 177:524–40. doi: 10.1016/j.cell.2019.03.016 PMC6538279 31002794

[5] Gitlin AD Shulman Z Nussenzweig MC. Clonal selection in the germinal centre by regulated proliferation and hypermutation. Nature. (2014) 509:637–40. doi: 10.1038/nature13300 PMC4271732 24805232

[6] Wishnie AJ Chwat-Edelstein T Attaway M Vuong BQ. BCR affinity influences T-B interactions and B cell development in secondary lymphoid organs. Front Immunol. (2021) 12:703918. doi: 10.3389/fimmu.2021.703918 34381455 PMC8350505

[7] Stebegg M Kumar SD Silva-Cayetano A Fonseca VR Linterman MA Graca L. Regulation of the germinal center response. Front Immunol. (2018) 9:2469. doi: 10.3389/fimmu.2018.02469 30410492 PMC6209676

[8] Nutt SL Hodgkin PD Tarlinton DM Corcoran LM. The generation of antibody-secreting plasma cells. Nat Rev Immunol. (2015) 15:160–71. doi: 10.1038/nri3795 25698678

[9] Muramatsu M Kinoshita K Fagarasan S Yamada S Shinkai Y Honjo T. Class switch recombination and hypermutation require activation-induced cytidine deaminase (AID), a potential RNA editing enzyme. Cell. (2000) 102:553–63. doi: 10.1016/S0092-8674(00)00078-7 11007474

[10] Dickerson SK Market E Besmer E Papavasiliou FN. AID mediates hypermutation by deaminating single stranded DNA. J Exp Med. (2003) 197:1291–6. doi: 10.1084/jem.20030481 PMC2193777 12756266

[11] Crotty S. T follicular helper cell biology: A decade of discovery and diseases. Immunity. (2019) 50:1132–48. doi: 10.1016/j.immuni.2019.04.011 PMC6532429 31117010

[12] Mesin L Ersching J Victora GD. Germinal center B cell dynamics. Immunity. (2016) 45:471–82. doi: 10.1016/j.immuni.2016.09.001 PMC5123673 27653600

[13] Linterman MA Pierson W Lee SK Kallies A Kawamoto S Rayner TF. Foxp3+ follicular regulatory T cells control the germinal center response. Nat Med. (2011) 17:975–82. doi: 10.1038/nm.2425 PMC3182542 21785433

[14] Wollenberg I Agua-Doce A Hernández A Almeida C Oliveira VG Faro J. Regulation of the germinal center reaction by Foxp3+ follicular regulatory T cells. J Immunol Baltim Md 1950. (2011) 187:4553–60. doi: 10.4049/jimmunol.1101328 21984700

[15] Clement RL Daccache J Mohammed MT Diallo A Blazar BR Kuchroo VK. Follicular regulatory T cells control humoral and allergic immunity by restraining early B cell responses. Nat Immunol. (2019) 20:1360–71. doi: 10.1038/s41590-019-0472-4 PMC6754271 31477921

[16] Sage PT Sharpe AH. The multifaceted functions of follicular regulatory T cells. Curr Opin Immunol. (2020) 67:68–74. doi: 10.1016/j.coi.2020.10.009 33176227 PMC7744352

[17] Lu Y Craft J. T follicular regulatory cells: choreographers of productive germinal center responses. Front Immunol. (2021) 12:679909. doi: 10.3389/fimmu.2021.679909 34177925 PMC8222975

[18] Gonzalez-Figueroa P Roco JA Papa I Núñez Villacís L Stanley M Linterman MA. Follicular regulatory T cells produce neuritin to regulate B cells. Cell. (2021) 184:1775–1789.e19. doi: 10.1016/j.cell.2021.02.027 33711260

[19] Ghosh S Leavenworth JW. Current advances in follicular regulatory T-cell biology. Crit Rev Immunol. (2022) 42:35–47. doi: 10.1615/CritRevImmunol.2022045746 37017287 PMC11034780

[20] Gutiérrez-González M Fahad AS Ardito M Nanaware P Lu L Normandin E. Human antibody immune responses are personalized by selective removal of MHC-II peptide epitopes. bioRxiv. (2021), 426750. doi: 10.1101/2021.01.15.426750v1

[21] Groot AD Martin W Rivera DS. Regulatory t cell epitopes, compositions and uses thereof(2009). Available online at: https://patents.google.com/patent/US20090018067A1/en (Accessed November 01, 2024).

[22] De Groot AS Moise L McMurry JA Wambre E Van Overtvelt L Moingeon P. Activation of natural regulatory T cells by IgG Fc-derived peptide “Tregitopes. Blood. (2008) 112:3303–11. doi: 10.1182/blood-2008-02-138073 PMC2569179 18660382

[23] Hsieh LE Sidney J Burns JC Boyle DL Firestein GS Altman Y. IgG epitopes processed and presented by igG+ B cells induce suppression by human thymic-derived regulatory T cells. J Immunol Baltim Md 1950. (2021) 206:1194–203. doi: 10.4049/jimmunol.2001009 PMC7939040 33579724

[24] Cousens LP Najafian N Mingozzi F Elyaman W Mazer B Moise L. *In vitro* and *in vivo* studies of IgG-derived Treg epitopes (Tregitopes): a promising new tool for tolerance induction and treatment of autoimmunity. J Clin Immunol. (2013) 33 Suppl 1:S43–49. doi: 10.1007/s10875-012-9762-4 PMC3538121 22941509

[25] De Groot AS Martin W. Reducing risk, improving outcomes: bioengineering less immunogenic protein therapeutics. Clin Immunol Orlando Fla. (2009) 131:189–201. doi: 10.1016/j.clim.2009.01.009 19269256

[26] Mattei AE Gutierrez AH Seshadri S Tivin J Ardito M Rosenberg AS. In silico methods for immunogenicity risk assessment and human homology screening for therapeutic antibodies. mAbs. (2024) 16:2333729. doi: 10.1080/19420862.2024.2333729 38536724 PMC10978032

[27] Jaffe DB Shahi P Adams BA Chrisman AM Finnegan PM Raman N. Functional antibodies exhibit light chain coherence. Nature. (2022) 611:352–7. doi: 10.1038/s41586-022-05371-z PMC9607724 36289331

[28] Lu Y Jiang R Freyn AW Wang J Strohmeier S Lederer K. CD4+ follicular regulatory T cells optimize the influenza virus-specific B cell response. J Exp Med. (2021) 218:e20200547. doi: 10.1084/jem.20200547 33326020 PMC7748821

[29] Cavazzoni CB Hanson BL Podestà MA Bechu ED Clement RL Zhang H. Follicular T cells optimize the germinal center response to SARS-CoV-2 protein vaccination in mice. Cell Rep. (2022) 38:110399. doi: 10.1016/j.celrep.2022.110399 35139367 PMC8806144

[30] Jerne NK. Towards a network theory of the immune system. Ann Immunol. (1974) 125C:373–89. 4142565

[31] Moise L Gutierrez AH Bailey-Kellogg C Terry F Leng Q Abdel Hady KM. The two-faced T cell epitope: examining the host-microbe interface with JanusMatrix. Hum Vaccines Immunother. (2013) 9:1577–86. doi: 10.4161/hv.24615 PMC3974887 23584251

[32] Moise L Gutierrez A Kibria F Martin R Tassone R Liu R. iVAX: An integrated toolkit for the selection and optimization of antigens and the design of epitope-driven vaccines. Hum Vaccines Immunother. (2015) 11:2312–21. doi: 10.1080/21645515.2015.1061159 PMC4635942 26155959

[33] De Groot AS Moise L Liu R Gutierrez AH Terry F Koita OA. Cross-conservation of T-cell epitopes: now even more relevant to (H7N9) influenza vaccine design. Hum Vaccines Immunother. (2014) 10:256–62. doi: 10.4161/hv.28135 PMC4185886 24525618

[34] Chen Y Mason GH Scourfield DO Greenshields-Watson A Haigh TA Sewell AK. Structural definition of HLA class II-presented SARS-CoV-2 epitopes reveals a mechanism to escape pre-existing CD4+ T cell immunity. Cell Rep. (2023) 42:112827. doi: 10.1016/j.celrep.2023.112827 37471227 PMC10840515

[35] De Groot AS Rosenberg AS Miah SMS Skowron G Roberts BJ Lélias S. Identification of a potent regulatory T cell epitope in factor V that modulates CD4+ and CD8+ memory T cell responses. Clin Immunol Orlando Fla. (2021) 224:108661. doi: 10.1016/j.clim.2020.108661 33412295

[36] De Groot AS Khan S Mattei AE Lelias S Martin WD. Does human homology reduce the potential immunogenicity of non-antibody scaffolds? Front Immunol. (2023) 14:1215939. doi: 10.3389/fimmu.2023.1215939 38022550 PMC10664710

[37] Cousens L Najafian N Martin WD De Groot AS. Tregitope: immunomodulation powerhouse. Hum Immunol. (2014) 75:1139–46. doi: 10.1016/j.humimm.2014.10.012 25454619

[38] Cousens LP Su Y McClaine E Li X Terry F Smith R. Application of IgG-derived natural Treg epitopes (IgG Tregitopes) to antigen-specific tolerance induction in a murine model of type 1 diabetes. J Diabetes Res. (2013) 2013:621693. doi: 10.1155/2013/621693 23710469 PMC3655598

[39] De Groot AS Skowron G White JR Boyle C Richard G Serreze D. Therapeutic administration of Tregitope-Human Albumin Fusion with Insulin Peptides to promote Antigen-Specific Adaptive Tolerance Induction. Sci Rep. (2019) 9:16103. doi: 10.1038/s41598-019-52331-1 31695065 PMC6834854

[40] Dembele M Tao S Massoud AH Miah SMS Lelias S De Groot AS. Tregitopes improve asthma by promoting highly suppressive and antigen-specific tregs. Front Immunol. (2021) 12:634509. doi: 10.3389/fimmu.2021.634509 33953711 PMC8089381

[41] van der Marel S Comijn EM Verspaget HW van Deventer S van den Brink GR Petry H. Neutralizing antibodies against adeno-associated viruses in inflammatory bowel disease patients: implications for gene therapy. Inflammation Bowel Dis. (2011) 17:2436–42. doi: 10.1002/ibd.21673 21370319

[42] Kedzierska AE Lorek D Slawek A Chelmonska-Soyta A. Tregitopes regulate the tolerogenic immune response and decrease the foetal death rate in abortion-prone mouse matings. Sci Rep. (2020) 10:10531. doi: 10.1038/s41598-020-66957-z 32601347 PMC7324366

[43] De Groot AS Desai AK Lelias S Miah SMS Terry FE Khan S. Immune tolerance-adjusted personalized immunogenicity prediction for pompe disease. Front Immunol. (2021) 12:636731. doi: 10.3389/fimmu.2021.636731 34220802 PMC8242953

[44] Benet ZL Marthi M Ke F Wu R Turner JS Gabayre JB. CCL3 promotes germinal center B cells sampling by follicular regulatory T cells in murine lymph nodes. Front Immunol. (2018) 9:2044. doi: 10.3389/fimmu.2018.02044 30271404 PMC6146081

[45] Sage PT Ron-Harel N Juneja VR Sen DR Maleri S Sungnak W. Suppression by TFR cells leads to durable and selective inhibition of B cell effector function. Nat Immunol. (2016) 17:1436–46. doi: 10.1038/ni.3578 PMC5502675 27695002

[46] Ke F Benet ZL Maz MP Liu J Dent AL Kahlenberg JM. Germinal center B cells that acquire nuclear proteins are specifically suppressed by follicular regulatory T cells. eLife. (2023) 12:e83908. doi: 10.7554/eLife.83908.sa2 36862132 PMC9981149

[47] Ke F Benet ZL Shelyakin P Britanova OV Gupta N Dent AL. Targeted checkpoint control of B cells undergoing positive selection in germinal centers by follicular regulatory T cells. Proc Natl Acad Sci U S A. (2024) 121:e2304020121. doi: 10.1073/pnas.2304020121 38261619 PMC10835130

[48] Davda J Declerck P Hu-Lieskovan S Hickling TP Jacobs IA Chou J. Immunogenicity of immunomodulatory, antibody-based, oncology therapeutics. J Immunother Cancer. (2019) 7:105. doi: 10.1186/s40425-019-0586-0 30992085 PMC6466770

[49] Benvenuto M Mattera R Masuelli L Tresoldi I Giganti MG Frajese GV. The crossroads between cancer immunity and autoimmunity: antibodies to self antigens. Front Biosci Landmark Ed. (2017) 22:1289–329. doi: 10.2741/4545 28199204

[50] Akkaya B Oya Y Akkaya M Al Souz J Holstein AH Kamenyeva O. Regulatory T cells mediate specific suppression by depleting peptide-MHC class II from dendritic cells. Nat Immunol. (2019) 20:218–31. doi: 10.1038/s41590-018-0280-2 PMC6402611 30643268

[51] Gu P Gao JF D’Souza CA Kowalczyk A Chou KY Zhang L. Trogocytosis of CD80 and CD86 by induced regulatory T cells. Cell Mol Immunol. (2012) 9:136–46. doi: 10.1038/cmi.2011.62 PMC4002813 22307040

[52] Botta D Fuller MJ Marquez-Lago TT Bachus H Bradley JE Weinmann AS. Dynamic regulation of T follicular regulatory cell responses by interleukin 2 during influenza infection. Nat Immunol. (2017) 18:1249–60. doi: 10.1038/ni.3837 PMC5679073 28892471

[53] Maceiras AR Almeida SCP Mariotti-Ferrandiz E Chaara W Jebbawi F Six A. T follicular helper and T follicular regulatory cells have different TCR specificity. Nat Commun. (2017) 8:15067. doi: 10.1038/ncomms15067 28429709 PMC5413949

[54] Ye J Ma N Madden TL Ostell JM. IgBLAST: an immunoglobulin variable domain sequence analysis tool. Nucleic Acids Res. (2013) 41:W34–40. doi: 10.1093/nar/gkt382 PMC3692102 23671333

[55] Weber CR Akbar R Yermanos A Pavlović M Snapkov I Sandve GK. immuneSIM: tunable multi-feature simulation of B- and T-cell receptor repertoires for immunoinformatics benchmarking. Bioinforma Oxf Engl. (2020) 36:3594–6. doi: 10.1093/bioinformatics/btaa158 PMC7334888 32154832

[56] Gupta NT Vander Heiden JA Uduman M Gadala-Maria D Yaari G Kleinstein SH. Change-O: a toolkit for analyzing large-scale B cell immunoglobulin repertoire sequencing data. Bioinforma Oxf Engl. (2015) 31:3356–8. doi: 10.1093/bioinformatics/btv359 PMC4793929 26069265

[57] McMurry JA Gregory SH Moise L Rivera D Buus S De Groot AS. Diversity of Francisella tularensis Schu4 antigens recognized by T lymphocytes after natural infections in humans: identification of candidate epitopes for inclusion in a rationally designed tularemia vaccine. Vaccine. (2007) 25:3179–91. doi: 10.1016/j.vaccine.2007.01.039 17291638

[58] Liu R Moise L Tassone R Gutierrez AH Terry FE Sangare K. H7N9 T-cell epitopes that mimic human sequences are less immunogenic and may induce Treg-mediated tolerance. Hum Vaccines Immunother. (2015) 11:2241–52. doi: 10.1080/21645515.2015.1052197 PMC4635734 26090577

