# Supplementary material for: Regulatory T cell epitope content in human antibodies decreases during maturation
Source: Front Immunol. 2025 Apr 17;16:1535826. doi: 10.3389/fimmu.2025.1535826 (PMC12043479; doi:10.3389/fimmu.2025.1535826)
Supplement: Supplementary Figure 1 — Comparison of Tregitope content among isotypes for antibodies with similar SHM. Box plots show Tregitope content in antibodies grouped by isotype and SHM. SHM was truncated to 15% and antibodies were binned based on SHM (5% range; bins: 0-5, 5-10, 10-15). Tregitope content was compared within each SHM bin using a Wilcoxon rank sum test. The median Tregitope content per isotype is shown at the bottom of each panel. Statistical significance is shown as *: p ≤ 0.05, **: p ≤ 0.01, ***: p ≤ 0.001, ****: p ≤ 0.0001. [file Presentation1.pdf]

Supplementary Material

1 Supplementary Tables and Figures

1.1 Supplementary Tables

Supplementary Table 1. Spearman ρ correlation between T cell epitope content subsets and SHM by isotype

Donor	Isotype	Number of antibodies	T cell epitope content	Tregitope content	JMX high content	JMX low content
1	IgA	28,471	-0.14	-0.32	-0.17	0.28
	IgG	16,032	-0.15	-0.36	-0.22	0.33
	IgM	80,332	-0.13	-0.24	-0.13	0.23
2	IgA	4,951	-0.15	-0.32	-0.24	0.38
	IgG	9,909	-0.13	-0.28	-0.25	0.37
	IgM	15,293	-0.08	-0.24	-0.17	0.37
3	IgA	11,955	-0.14	-0.33	-0.23	0.31
	IgG	10,648	-0.15	-0.37	-0.23	0.31
	IgM	20,995	-0.06	-0.25	-0.19	0.35
4	IgA	21,643	-0.14	-0.30	-0.22	0.33
	IgG	13,665	-0.10	-0.29	-0.21	0.32
	IgM	14,645	-0.12	-0.24	-0.17	0.29

Spearman ρ correlation coefficients are colored from low (blue) to high (red). P-values were below 0.01.

Supplementary Table 2. Comparison of T cell epitope content subsets between switched memory B cells and plasmablasts (Pb)

Donor	SHM bin	Number		Tregitope content					JMX high content					JMX low content				
		Memory	Pb	Median Memory	Median Pb	Ratio medians	p-value	$-\log_{10}$ p-value	Median Memory	Median Pb	Ratio medians	p-value	$-\log_{10}$ p-value	Median Memory	Median Pb	Ratio medians	p-value	$-\log_{10}$ p-value
1	0-5	4,391	330	10.12	9.07	1.116	**0.04984**	**1.302**	17.1	16.36	1.045	0.46263	0.335	14.56	13.68	1.064	0.05057	1.296
3	0-5	2,633	79	12.29	10.4	1.182	0.06597	1.181	17.46	17.45	1.001	0.70193	0.154	18.37	18.18	1.010	0.58569	0.232
4	0-5	3,339	304	10.32	8.55	1.207	**0.05000**	**1.301**	19.47	19.63	0.992	0.80824	0.092	15.9	16.36	0.972	0.51403	0.289
1	5-10	15,697	540	8.41	8.4	1.001	0.47749	0.321	15.2	15.41	0.986	0.55136	0.259	17.51	16.98	1.031	**0.02378**	**1.624**
3	5-10	8,124	253	10.34	8.7	1.189	**0.00160**	**2.795**	15.83	16.31	0.971	0.57883	0.237	21.41	20.68	1.035	0.25290	0.597
4	5-10	12,736	1,191	8.54	8.13	1.05	**0.00003**	**4.602**	18.02	17.41	1.035	0.06680	1.175	19.07	19.02	1.003	0.66004	0.180
1	10-15	14,849	464	6.41	6.17	1.039	**0.00747**	**2.127**	13.95	14.54	0.959	0.97924	0.009	19.81	19.98	0.991	**0.03871**	**1.412**
3	10-15	6,820	454	8.01	6.96	1.151	**0.00037**	**3.431**	14.17	14.28	0.992	0.91348	0.039	23.78	24.56	0.968	0.07968	1.099
4	10-15	11,973	1,671	6.68	6.15	1.086	**0.0000***	**6.000**	16.28	15.51	1.050	**0.00001**	**5.046**	21.64	21.75	0.995	0.54346	0.265
1	15-20	6,738	393	4.48	4.37	1.025	0.49776	0.303	13.06	12.92	1.011	0.51179	0.291	21.43	22.73	0.943	**0.02830**	**1.548**
3	15-20	3,327	376	6.24	4.92	1.268	**0.01201**	**1.920**	13.15	12.07	1.089	**0.04416**	**1.355**	25.8	26.74	0.965	**0.02254**	**1.647**
4	15-20	5,404	1,122	5.76	5.76	1.000	0.57278	0.242	14.6	13.78	1.060	**0.00070**	**3.154**	23.95	23.52	1.018	0.20653	0.685

For each T cell epitope content subset, median values are colored from blue (low) to red (high).
Ratios>1 and significant are colored orange. Ratios<1 and significant are colored light blue.
Statistically significant p-values (≤0.05) and $-\log_{10}$ p-values (≥1.301) are shown in bold font.
*P-value = 0; set to 0.000001 to calculate $-\log_{10}$ p-value.

Supplementary Table 3. HLA-DR binding of Tregitopes and variants with reduced binding likelihood and human cross-conservation

Label	Version	Selection criteria	Expected HLA-DR binding	Expected Treg function	Sequence[a]	EMX hits[b]	JMX score[b]	HLA-DRB1 (IC_{50} nM)				
								*01:01 (d2)	*03:01 (d4)	*04:01 (d3)[c]	*11:01 (d3)	*15:01 (d2,d4)
Treg9A	Original	Tregitope	Promiscuous	Normal	GGLVQPGGSLRLSCAASGFTF	11	27.64	704	819	3,222	221	289
Treg9Av_rb	Variant	Reduced binding likelihood; EMX low	Reduced	Reduced; decreased HLA binding affinity	GGL**A**QPGGSLRLSC**EV**S**NV**T**S**	0	0.00	non-binder	non-binder	896	non-binder	22,918
Treg9Av_rxc	Variant	Reduced human cross-conservation; JMX low	Promiscuous	Reduced; decreased cognate Treg activation	GGL**A**QPGGSLRLSC**TP**SGF**I**F	7	0.14	non-binder	61,559	125	43,530	4,612
Treg88	Original	Tregitope	Promiscuous	Normal	NTLYLQMNSLRAEDTA	15	22.40	200	2,661	112	299	2,140
Treg88v_rb	Variant	Reduced binding likelihood; EMX low	Reduced	Reduced; decreased HLA binding affinity	NT**AS**L**H**M**HD**LR**P**ED**SG**	0	0.00	non-binder	non-binder	non-binder	non-binder	non-binder
Treg88v_rxc	Variant	Reduced human cross-conservation; JMX low	Promiscuous	Reduced; decreased cognate Treg activation	N**S**L**F**L**H**M**DN**LRAED**S**A	10	0.00	168	7,731	85	755	4,469

[a]Amino acid differences compared to original Tregitope sequences are shown in red font.
[b]EpiMatrix (EMX) hits and JanusMatrix (JMX) score calculated for all the HLA-DR alleles expressed by donors 2, 3, and 4 (d2, d3, and d4) (HLA-DRB1*01:01, *03:01, *04:04, *11:01, *13:01, *15:01, and *16:01).
[c]HLA-DRB1*04:01 used instead of *04:04 (expressed by donor 3). These alleles share similar binding peptide side-chain preferences for binding pockets.

Non-binder (IC_{50} >100,000nM)
Low affinity (IC_{50} 10,000-100,000nM)
Moderate affinity (IC_{50} 1,000-10,000nM)
High affinity (IC_{50} 100-1,000nM)
Very high affinity (IC_{50} <100nM)

1.2 Supplementary Figures

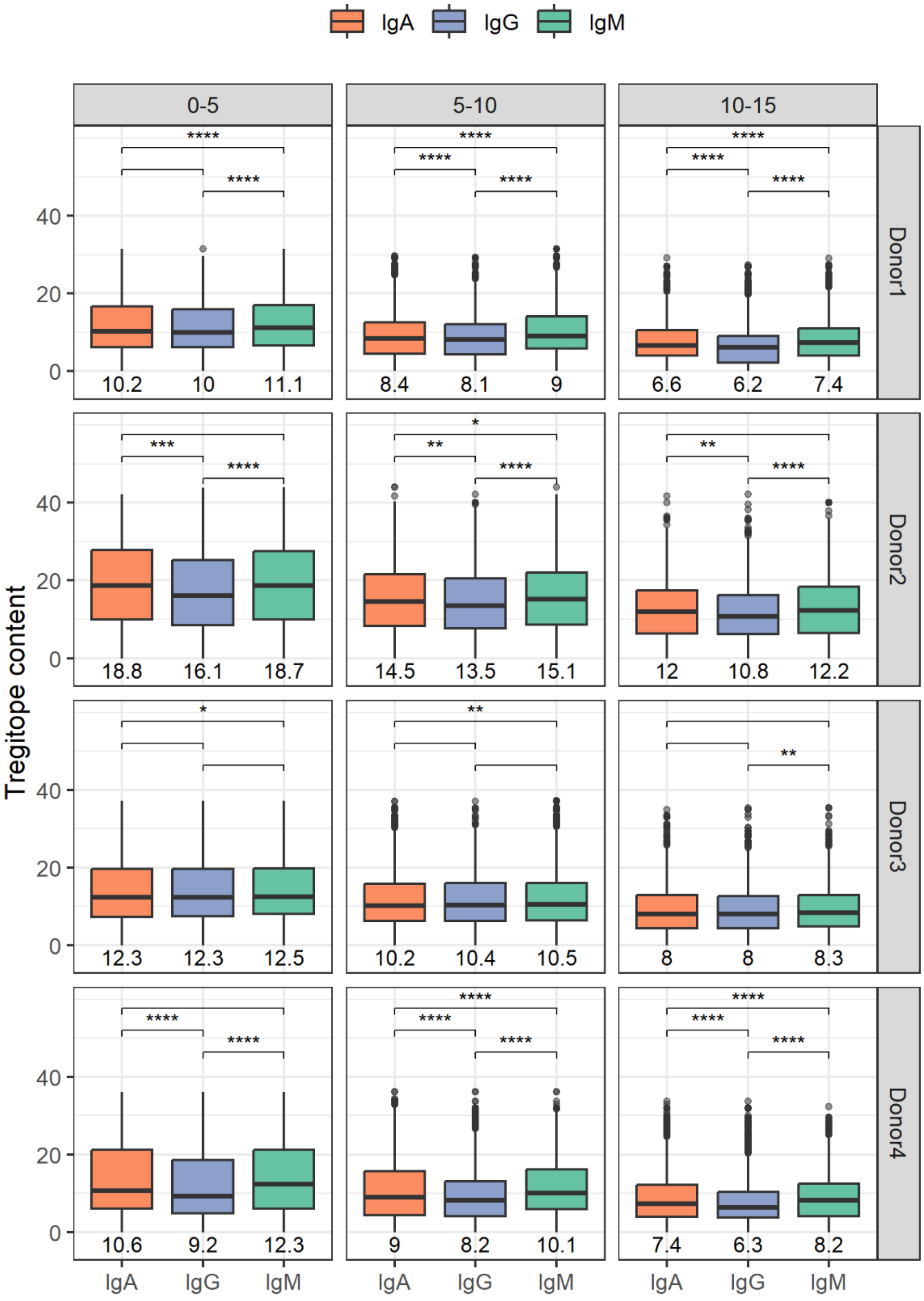

Supplementary Figure 1. Comparison of Tregitope content among isotypes for antibodies with similar SHM. Box plots show Tregitope content in antibodies grouped by isotype and SHM. SHM was truncated to 15% and antibodies were binned based on SHM (5% range; bins: 0-5, 5-10, 10-15). Tregitope content was compared within each SHM bin using a Wilcoxon rank sum test. The median Tregitope content per isotype is shown at the bottom of each panel. Statistical significance is shown as *: p≤0.05, **: p≤0.01, ***: p≤0.001, ****: p≤0.0001.

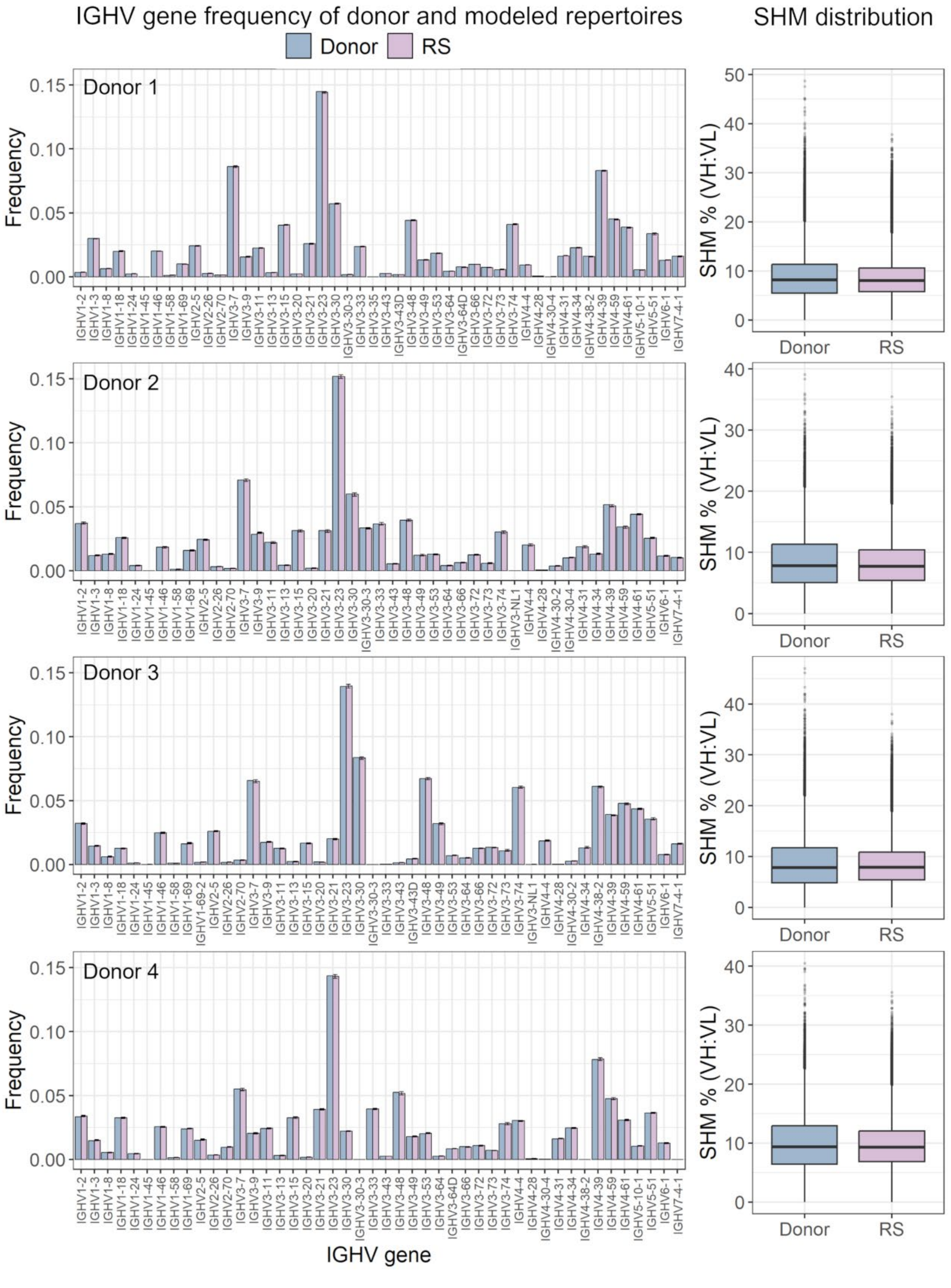

Supplementary Figure 2. Comparison between donor and modeled repertoires. (A) VH gene usage (frequency) and (B) SHM distribution comparison between donor and modeled repertoire using donor-specific replacement-silent (RS) SHM models. We simulated 10 donor-specific antibody repertoires that matched the V-gene frequencies, 5-mer DNA targeting patterns, and SHM distribution in each individual donor repertoire.

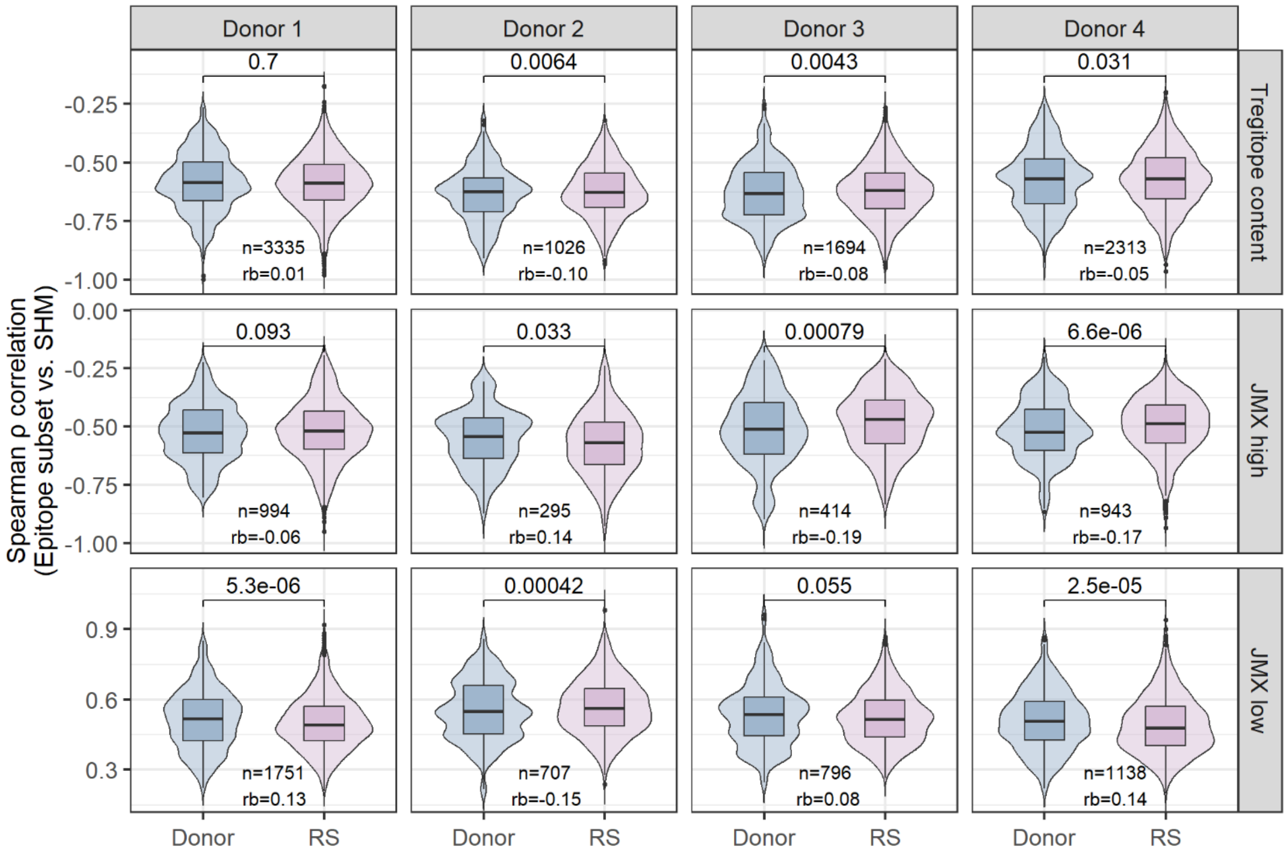

Supplementary Figure 3. Comparison of T cell epitope content changes with SHM between donor and modeled class-switched repertoires. The Spearman ρ correlation coefficients of V-gene pairs with significant correlation between T cell epitope content subsets and SHM for the donor and replacement-silent (RS) switched modeled repertoires were compared using paired samples Wilcoxon rank sum test. V-gene pairs that were significant for both the donor and the model repertoires were included in the comparison. For each comparison, p-values, the cumulative number of compared pairs between donor repertoires and each of the 10 RS models and rank-biserial coefficients (rb) are shown. Effect sizes were defined using rank-biserial correlations; values range from -1, indicating complete dominance of the model sample (all the Spearman ρ correlation coefficients of the model are larger than all the coefficients of the donor), to +1, indicating complete dominance of the donor sample (all the coefficients of the donor are larger than all the coefficients of the model). Negative rb for Tregitope and JMX high indicates that a larger proportion of V-gene pairs from the donors' repertoires had lower (negative) Spearman correlation coefficients compared to their modeled repertoires. Positive rb for JMX low indicates that a larger proportion of V-gene pairs from the donors' repertoires had higher (positive) Spearman correlation coefficients compared to their modeled repertoires.

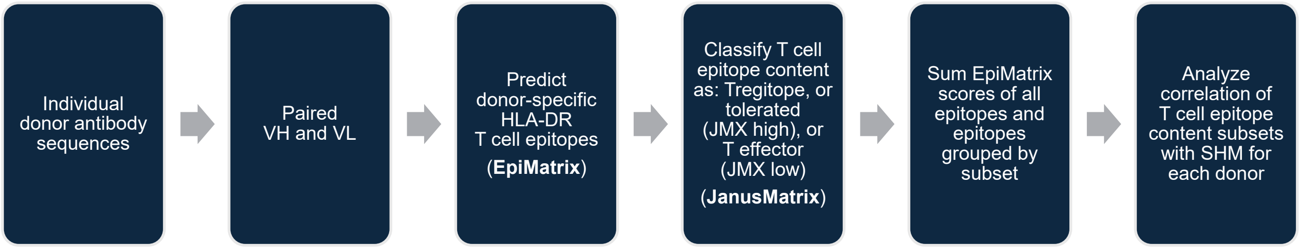

Supplementary Figure 4. Prediction and analysis pipeline of personalized T cell epitope content.
